# How Has the Age-Related Process of Overweight or Obesity Development Changed over Time? Co-ordinated Analyses of Individual Participant Data from Five United Kingdom Birth Cohorts

**DOI:** 10.1371/journal.pmed.1001828

**Published:** 2015-05-19

**Authors:** William Johnson, Leah Li, Diana Kuh, Rebecca Hardy

**Affiliations:** 1 Medical Research Council Unit for Lifelong Health and Ageing at University College London, London, United Kingdom; 2 University College London Institute of Child Health, London, United Kingdom; University of Oxford, UNITED KINGDOM

## Abstract

**Background:**

There is a paucity of information on secular trends in the age-related process by which people develop overweight or obesity. Utilizing longitudinal data in the United Kingdom birth cohort studies, we investigated shifts over the past nearly 70 years in the distribution of body mass index (BMI) and development of overweight or obesity across childhood and adulthood.

**Methods and Findings:**

The sample comprised 56,632 participants with 273,843 BMI observations in the 1946 Medical Research Council National Survey of Health and Development (NSHD; ages 2–64 years), 1958 National Child Development Study (NCDS; 7–50), 1970 British Cohort Study (BCS; 10–42), 1991 Avon Longitudinal Study of Parents and Children (ALSPAC; 7–18), or 2001 Millennium Cohort Study (MCS; 3–11). Growth references showed a secular trend toward positive skewing of the BMI distribution at younger ages. During childhood, the 50th centiles for all studies lay in the middle of the International Obesity Task Force normal weight range, but during adulthood, the age when a 50th centile first entered the overweight range (i.e., 25–29.9 kg/m^2^) decreased across NSHD, NCDS, and BCS from 41 to 33 to 30 years in males and 48 to 44 to 41 years in females. Trajectories of overweight or obesity showed that more recently born cohorts developed greater probabilities of overweight or obesity at younger ages. Overweight or obesity became more probable in NCDS than NSHD in early adulthood, but more probable in BCS than NCDS and NSHD in adolescence, for example. By age 10 years, the estimated probabilities of overweight or obesity in cohorts born after the 1980s were 2–3 times greater than those born before the 1980s (e.g., 0.229 [95% CI 0.219–0.240] in MCS males; 0.071 [0.065–0.078] in NSHD males). It was not possible to (1) model separate trajectories for overweight and obesity, because there were few obesity cases at young ages in the earliest-born cohorts, or (2) consider ethnic minority groups. The end date for analyses was August 2014.

**Conclusions:**

Our results demonstrate how younger generations are likely to accumulate greater exposure to overweight or obesity throughout their lives and, thus, increased risk for chronic health conditions such as coronary heart disease and type 2 diabetes mellitus. In the absence of effective intervention, overweight and obesity will have severe public health consequences in decades to come.

## Abstract 2

In a longitudinal analysis, William Johnson and colleagues examine how individual lifetime BMI trajectories among white citizens of the UK have changed from 1946 to 2014.

## Editors' Summary

**Background:**

Overweight and obesity are major threats to global health. The global prevalence of obesity (the proportion of the world's population that is obese) has more than doubled since 1980; 13% of the adult population, or 0.6 billion people, are now classified as obese, while an additional 1.3 billion adults are overweight. Both classifications are determined by body mass index (BMI), which is calculated by dividing a person's weight in kilograms by the square of their height in meters. Obese individuals have a BMI of 30 kg/m2 or more, while overweight individuals have a BMI of 25–30 kg/m2. BMI values above 25 kg/m2 increase the risk of developing non-communicable diseases (NCDs), including cardiovascular diseases, cancers and diabetes. Each year, NCDs kill 38 million people (including 28 million people in low- and middle-income countries and 9 million people under 60 years of age), thereby accounting for more than 75% of the world's annual deaths.

In the United Kingdom, studies report that roughly one quarter of adults are obese, and a further third or more are overweight. This “obesity epidemic” extends to children; according to the National Child Measurement Programme for England (NCMP), about 9% of 4–5-year-olds and 19% of 10–11-year-olds were obese in 2013. In parallel, the UK has not seen the improvements in child and young adult mortality seen in comparable European states.

**Why Was This Study Done?:**

Cross-sectional surveys in the UK, United States, and elsewhere have documented the obesity epidemic, but longitudinal data—drawn from periodic BMI measurements from individuals over their lifetimes—are needed to clarify the time course, or trajectory, of overweight and obesity. Longitudinal data can answer practical questions important for designing health policy interventions. Is the age at which individuals develop overweight or obesity changing over time? In which individuals are the greatest increases in BMI occurring? The authors leveraged longitudinal data from five birth cohort studies (studies that follow a selected group of individuals born during a short window of time), incepted in 1946, 1958, 1970, 1991, and 2001. These large cohort projects were funded by the UK government for the purpose of providing data for long-term health analyses such as this one; in total, the current study’s included sample comprised 56,632 participants with 273,843 BMI observations from participants aged 2 through 64.

**What Did the Researchers Do and Find?:**

The present study aimed to investigate (1) shifts from the 1940s to the 2000s in the distribution of BMI across age and (2) shifts over the same period in the probability of developing overweight or obesity across age. For each of the five cohorts, subdivided by sex and childhood versus adulthood (thus, a total of 20 datasets), the authors applied statistical models to produce trajectories for each BMI centile (subset that results from dividing the distribution of BMI measurements into 100 groups with equal frequency; here, the 90th centile is the group for which 90% of the relevant population has lower BMI). They then investigated secular trends (long-term, non-periodic variations) at different centiles of the BMI distribution. For example, by comparing the trajectories of the 50th centile for adult males across the five cohorts, the researchers could see how the age at which BMI values reached the obese range varied between eras among this group.

The data revealed that most of the between-cohort, and thus between-era, increases in BMI took place in the highest centiles, indicating that overall gains in BMI mainly comprised very high BMI individuals carrying even more weight. Across the 1946, 1958, and 1970 cohorts, the age at which the 50th centile of adults entered the overweight range decreased from 41 to 33 to 30 years in males and 48 to 44 to 41 years in females. The probabilities of overweight and obesity across adulthood also increased. While children in the 50th BMI centile have remained at normal weight through the decades, the overall childhood probability of developing overweight or obesity has increased 2–3-fold from before to after the 1980s.

**What Do These Findings Mean?:**

These findings describe the changing pattern of age-related progression of overweight and obesity from early childhood in white citizens of the UK. The findings may not be generalizable because other populations have distinct genetic predispositions, environmental exposures, and access to health care. In addition, the accuracy of the findings may be affected by differences between cohorts in how weight and height (and thus BMI) were measured. Nevertheless, these findings—in particular, the increased risk of overweight and obesity at younger ages—suggest that compared to previous generations, current and future generations will accumulate greater overweight or obesity exposure across their lives, likely resulting in increased risk for NCDs. Further research is now needed to determine whether lifestyle factors in the UK have affected the trajectory of BMI and to discover the extent to which these shifting weight trajectories have contributed to morbidity and mortality.

**Additional Information:**

This list of resources contains links that can be accessed when viewing the PDF on a device or via the online version of the article at http://dx.doi.org/10.1371/journal.pmed.1001828.

- The World Health Organization provides information on obesity and non-communicable diseases around the world (in several languages)
- The UK National Health Service Choices website also provides detailed information about obesity and a link to a personal story about losing weight
- The International Obesity Taskforce provides information about the global obesity epidemic
- The US Centers for Disease Control and Prevention provides information on non-communicable diseases around the world and on overweight and obesity and diabetes (including some information in Spanish)
- The US Department of Agriculture's ChooseMyPlate.gov website provides a personal healthy eating plan
- The Weight-control Information Network is an information service provided for the general public and health professionals by the US National Institute of Diabetes and Digestive and Kidney Diseases (in English and Spanish)
- MedlinePlus has links to further information about obesity (in English and Spanish)

## Introduction

The obesity epidemic is a daunting public health threat, even in high-income countries with good infrastructure for education and health care. In the United Kingdom, the projected total cost to the National Health Service (NHS) is estimated to be £22.9 billion per year by 2050 [1]. The 2012 Health Survey for England (HSE) reported that 25% of adults were obese and a further 42% of males and 32% of females were overweight, according to body mass index (BMI) [2]. Similar results have been published for Scotland, Wales, and Northern Ireland [3–5], and worryingly high prevalence rates exist in most other high-income countries outside of the UK [6]. Children in such settings have not been exempt from the epidemic [6]. The 2012–2013 National Child Measurement Programme for England (NCMP) reported that 9.3% of 4–5 year olds and 18.9% of 10–11 year olds were obese, for example [7]. This is particularly concerning given that childhood obesity tends to lead to adulthood obesity, and this tracking is often accompanied by the development of other cardiovascular risk factors, such as dyslipidaemia, hyperglycaemia, hypertension, and vascular inflammation [8]. Obesity rates are, therefore, associated with rates of adiposity-related conditions, such as coronary heart disease (CHD) and type 2 diabetes mellitus [9–11].

Cross-sectional surveys in the UK and elsewhere estimate the scale of the problem, but longitudinal data are needed to provide information on the age-related process of obesity development, such as the ages when risk is increasing rapidly. Such trajectories are related to disease processes [12–18]. Some surveys in the UK have been repeated over time, thereby allowing demonstration of relatively recent (since 1991 in HSE and 2005 in NCMP) increases in prevalence rates [2,7], while longer-term secular trends have been published in other high-income countries, for example, using data from the National Health and Nutrition Examination Surveys (NHANES) in the United States [19]. Such publications do not, however, tell us how the process of obesity development across childhood and adulthood has changed over time in response to environment changes, including those in the behavioural, sociocultural, and economic landscape. This information would be useful to identify appropriate ages and patterns of overweight or obesity development for targeted prevention or intervention and to provide insight into the possible aetiological factors responsible.

The UK has uniquely invested in a series of four nationally representative birth cohort studies that could be used to investigate the development of obesity across age and time [20]. Li et al. published age-related mean BMI trajectories in the two oldest studies, the 1946 Medical Research Council National Survey of Health and Development (1946 NSHD) and the 1958 National Child Development Study (1958 NCDS); the trajectories were similar in childhood but diverged in early adulthood such that the more recently born cohort gained an additional 0.06 kg/m^2^ per year [21]. The rest of our knowledge is based on studies in which data have been treated cross-sectionally or studies that are (1) not representative of the UK population, (2) span only a small part of the life course, and/or (3) describe only a short-term trend [18,22–24]. Studies conducted outside of the UK, mainly in the US, are similarly limited [25–27]. Furthermore, all of these published cohort studies have focused on mean BMI trajectories, despite the predominant discourse based on studies in diverse settings around the world being that the obesity epidemic reflects shifts at the upper end of the distribution [28–33].

The present study utilises the extensive longitudinal data on BMI in the UK birth cohort studies, with the aims to investigate (1) shifts over time in the distribution of BMI across age and (2) shifts over time in the development of overweight or obesity across age.

## Methods

### Study Samples

The 1946 NSHD is based on a sample (*n* = 5,362) of all singleton births in one week in March 1946 in England, Scotland, and Wales [34,35]. The 1958 NCDS is based on 17,638 people born in one week in March 1958 in England, Scotland, and Wales; 920 immigrants born in the same week were incorporated during childhood [36]. A similar strategy was used in the 1970 British Cohort Study (1970 BCS), which is based on 17,287 people born in one week in April 1970, with the addition of 1,814 individuals who were (1) born in Northern Ireland and included only in the birth sweep, (2) immigrants who were incorporated into the study in childhood, or (3) never took part in any sweep [37]. The 1991 Avon Longitudinal Study of Parents and Children (1991 ALSPAC) is based on 15,444 births to women living in the defunct county of Avon in England with an expected delivery date between April 1991 and December 1992; this number includes 713 people who were enrolled during childhood recruitment drives [38–40]. A fully searchable data dictionary is available for the 1991 ALSPAC study at http://www.bris.ac.uk/alspac/researchers/data-access/data-dictionary. This study was used to fill a 30-year gap in which no nationally representative study was initiated. Finally, the 2001 Millennium Cohort Study (2001 MCS) is based on 18,818 people born between September 2000 and January 2002 who were living in England, Scotland, Wales, or Northern Ireland at age 9 months [41]. All of the studies have received ethical approval and obtained informed parental and/or participant consent; this information is available from the study websites and/or cohort profiles [34–41].

The inclusion criteria for the present study excluded groups likely to have particularly different BMI values, thereby ensuring each study sample comprised a comparable composition of people. The criteria were (1) part of the original cohort (i.e., not an immigrant), (2) white race, (3) singleton birth, and (4) survival to at least age 9 months in the 2001 MCS; age 1 year in the 1946 NSHD, 1958 NCDS, and 1991 ALSPAC; and age 5 years in the 1970 BCS. Each study determined race/ethnicity differently and not always consistently across all sweeps; we use “white” as it best captures everyone in our sample (i.e., everyone was white British in the 1946 NSHD, while in the 1958 NCDS, 1970 BCS, 1991 ALSPAC, and 2001 MCS the samples were restricted to the responses “European/Caucasian,” “European/UK,” “White,” and “White,” respectively). Participants were not excluded on the basis of missing data for these variables, but were required to have sex recorded and at least one observation of BMI. Sample selection is shown in S1 Fig and sample sizes are shown in Table 1.

**Table 1:** Description of BMI data in the five UK birth cohort studies.

			Male					Female			
	Sweep		BMI (kg/m^2^)	Thinness^a^	Overweight^a^	Obesity^a^		BMI (kg/m^2^)	Thinness^a^	Overweight^a^	Obesity^a^
	Target age (date)	*n*	Median (IQR)	%	%	%	*n*	Median (IQR)	%	%	%
**1946 NSHD**	2 (1948)	2,046	17.7 (16.3, 19.2)	7.7	16.8	17.0	1,794	17.2 (16.1, 18.8)	7.2	21.8	14.9
2,598 males	4 (1950)	2,198	16.2 (15.3, 17.2)	10.5	16.5	6.1	1,986	15.9 (14.9, 17.1)	9.8	15.8	4.8
2,359 females	6 (1952)	2,050	15.9 (15.0, 16.7)	6.7	9.0	0.8	1,841	15.6 (14.8, 16.5)	8.2	10.3	1.3
	7 (1953)	2,057	15.8 (14.9, 16.6)	5.9	6.2	0.4	1,920	15.5 (14.7, 16.5)	9.6	7.4	1.1
	11 (1957)	2,050	16.9 (15.9, 18.1)	9.0	6.6	0.8	1,887	17.0 (15.7, 18.7)	12.5	8.5	1.8
	15 (1961)	1,881	19.3 (18.0, 20.8)	8.2	7.4	0.8	1,700	20.3 (18.6, 22.1)	8.8	11.0	1.7
	20 (1966)	1,802	22.5 (20.9, 24.0)	2.3	12.8	1.2	1,629	21.4 (19.8, 23.1)	8.0	8.7	1.7
	26 (1972)	1,822	23.1 (21.5, 25.1)	1.8	23.0	2.6	1,782	21.8 (20.2, 23.8)	5.3	13.9	2.8
	36 (1982)	1,631	24.6 (22.7, 26.7)	1.2	37.8	6.2	1,618	22.6 (20.9, 25.1)	3.7	18.5	7.1
	43 (1989)	1,612	25.3 (23.3, 27.7)	0.6	44.9	10.4	1,595	24.0 (22.1, 27.1)	1.6	25.8	13.8
	53 (1999)	1,451	27.0 (24.7, 29.7)	0.3	49.2	22.7	1,494	26.2 (23.7, 30.1)	0.3	36.3	25.8
	60–64 (2006–2010)	1,059	27.6 (25.0, 30.3)	0.3	46.7	28.1	1,155	26.9 (24.2, 31.0)	1.0	37.0	30.2
**1958 NCDS**	7 (1965)	6,499	15.8 (15.0, 16.7)	8.9	6.6	1.3	6,068	15.6 (14.6, 16.7)	9.7	8.7	2.2
7,927 males	11 (1969)	5,931	16.8 (15.8, 18.2)	12.4	6.7	1.3	5,687	17.1 (15.8, 18.9)	16.0	9.0	1.4
7,514 females	16 (1974)	5,194	19.8 (18.5, 21.4)	10.3	6.8	1.5	4,911	20.6 (19.0, 22.5)	9.9	10.3	1.6
	23 (1981)	5,680	22.7 (21.2, 24.5)	2.4	17.7	2.4	5,732	21.6 (20.1, 23.5)	6.4	11.6	3.1
	33 (1991)	5,006	25.1 (23.1, 27.5)	1.0	40.4	10.9	4,982	23.4 (21.5, 26.4)	3.4	23.2	11.8
	42 (2000)	5,069	26.0 (23.9, 28.5)	0.5	46.4	15.6	5,195	24.1 (22.0, 27.5)	1.7	26.6	15.4
	44 (2002)	4,249	27.3 (25.0, 30.1)	0.3	49.6	25.6	4,305	25.7 (23.1, 29.7)	0.8	32.8	23.5
	50 (2008)	3,833	27.4 (24.9, 30.4)	0.3	46.6	27.9	3,814	25.7 (22.9, 29.5)	1.3	32.9	22.9
**1970 BCS**	10 (1980)	5,738	16.4 (15.5, 17.7)	10.5	6.3	0.2	5,443	16.7 (15.5, 18.3)	12.1	10.3	0.5
7,111 males	16 (1986)	3,398	20.4 (19.0, 22.3)	10.1	9.3	1.9	3,868	20.9 (19.3, 23.0)	10.7	11.3	1.6
6,781 females	26 (1996)	2,322	24.1 (22.1, 26.1)	1.1	29.9	6.4	4,324	22.3 (20.7, 24.8)	4.2	16.9	6.6
	30 (2000)	4,796	25.1 (23.0, 27.6)	0.9	39.8	11.5	5,072	23.2 (21.1, 26.3)	3.2	21.9	11.1
	34 (2004)	4,107	26.0 (23.7, 28.7)	0.6	43.2	17.6	4,398	24.0 (21.6, 27.4)	2.2	25.4	15.5
	42 (2012)	3,907	26.8 (24.4, 29.8)	0.5	44.7	23.8	4,037	24.9 (22.3, 28.8)	1.8	29.0	20.3
**1991 ALSPAC**	7 (1998)	3,693	15.7 (14.9, 16.8)	7.8	9.2	2.5	3,567	15.9 (14.9, 17.3)	7.2	13.1	4.0
4,461 males	8 (1999)	3,048	16.5 (15.5, 17.8)	4.4	12.3	3.4	3,017	16.8 (15.5, 18.5)	4.4	17.5	4.6
4,404 females	9 (2000)	3,360	16.8 (15.6, 18.7)	7.7	13.5	3.6	3,412	17.3 (15.8, 19.4)	8.0	18.0	4.5
	10 (2001)	3,298	17.3 (15.9, 19.5)	7.1	14.4	4.1	3,338	17.7 (16.1, 20.0)	8.4	17.2	4.9
	11 (2002)	3,132	18.0 (16.5, 20.5)	7.6	16.2	4.4	3,207	18.6 (16.8, 21.2)	8.9	18.6	4.6
	13 (2004)	2,939	18.7 (17.1, 21.1)	7.9	16.0	4.3	3,016	19.4 (17.6, 21.9)	9.3	16.9	3.9
	14 (2005)	2,699	19.2 (17.7, 21.4)	8.0	13.4	3.9	2,761	20.1 (18.3, 22.4)	9.3	15.8	3.8
	15 (2006)	2,289	20.4 (18.8, 22.5)	6.5	13.6	3.9	2,537	21.1 (19.4, 23.5)	8.2	15.0	4.8
	18 (2009)	1,950	21.8 (20.0, 24.3)	8.2	15.9	5.9	2,487	22.0 (20.1, 24.8)	7.5	16.7	7.4
**2001 MCS**	3 (2004)	5,726	16.8 (16.0, 17.8)	3.0	18.6	5.1	5,625	16.6 (15.7, 17.5)	3.9	19.6	5.1
6,897 males	5 (2006)	6,114	16.2 (15.4, 17.1)	3.5	14.5	4.7	5,846	16.1 (15.2, 17.1)	3.3	18.3	5.8
6,580 females	7 (2008)	5,552	16.2 (15.2, 17.4)	4.7	12.7	4.9	5,399	16.3 (15.2, 17.7)	4.8	16.9	6.2
	11 (2012)	5,169	18.1 (16.5, 20.6)	5.1	18.7	6.0	5,037	18.7 (16.8, 21.5)	6.4	22.6	6.7

BMI: Body Mass Index, IOTF: International Obesity Task Force, IQR: Inter-Quartile Range, UK: United Kingdom, NSHD: Medical Research Council National Survey of Health and Development, NCDS National Child Development Study, BCS: British Cohort Study, ALSPAC: Avon Longitudinal Study of Parents and Children, MCS: Millennium Cohort Study

^a^Thinness, overweight, and obesity between 2–18 years of age were defined according to the IOTF cut-offs, which are centiles that link with the adulthood cut-offs at age 18 years (e.g., the 90.5th IOTF centile is used to define overweight in boys as this centile equals 25 kg/m^2^, the adulthood cut-off, at age 18 years).

### Anthropometry

Weight and height were assessed at data collection sweeps at target ages of 2, 4, 6, 7, 11, 15, 20, 26, 36, 43, 53, and 60–64 years in the 1946 NSHD; 7, 11, 16, 23, 33, 42, 44, and 50 years in the 1958 NCDS; 10, 16, 26, 30, 34, and 42 years in the 1970 BCS; 7, 8, 9, 10, 11, 13, 14, 15, and 18 years in the 1991 ALSPAC; and 3, 5, 7, and 11 years in the 2001 MCS. S1 Table summarises the main differences in measurement protocols and S2 Table describes the main steps used to make the anthropometry as comparable as possible.

### Statistical Analysis

WJ and RH determined which analyses to perform and include in the present paper in July 2013 after discussing options with all co-authors.

BMI was computed as weight (kg)/height (m)^2^, and thinness, overweight, and obesity were defined according to International Obesity Task Force (IOTF) cut-offs during childhood and standard cut-offs of 18.5, 25, and 30 kg/m^2^ during adulthood [42,43]. The IOTF cut-offs are centiles spanning 2–18 years of age that link with the adulthood cut-offs at age 18 years (e.g., the 90.5th IOTF centile is used to define overweight in boys as this centile equals 25 kg/m^2^, the adulthood cut-off, at age 18 years), thereby avoiding artificial change in prevalence during the transition to adulthood.

The Lambda-Mu-Sigma (LMS) method was used to summarise the distribution of BMI across age in sex, study, and childhood (ages 2–18 years) versus adulthood (ages 20–64 years) stratified models [44]. Briefly, the LMS method models variation in size across age as a function of three curves: (1) the lambda (L) curve describes the Box-Cox power needed to remove skewness, (2) the mu (M) curve describes the median, and (3) the sigma (S) curve describes the coefficient of variation. With these three curves it is possible to compute any centile, thereby allowing investigation of secular trends at different centiles of the BMI distribution. The three curves are fitted as cubic splines, with Equivalent Degrees of Freedom (EDF) governing their complexity. Models were built by choosing the EDF for M, then S, then L, with the aim to make EDF for M > EDF for S > EDF for L, before checking whether or not to use rescaled age. A change in the Bayesian Information Criterion (BIC) greater than 10 indicated improved model fit [45]. Model choice was also guided by visual inspection of the centiles against the observed data. Differences between the expected and observed percentage of participants with BMI above select centiles were investigated in the finals models. Common centiles used in the UK (98th, 91st, 50th, 9th, and 2nd) for each study were overlaid in centile and sex-specific plots.

Sex- and study-stratified binary logistic multilevel models (observations at level one nested within individuals at level two) were used to describe weight status trajectories, assuming missing data were at random. Overweight and obesity were combined as there were few cases of overweight or obesity at early sweeps in older studies (e.g., there were only 8 obese boys in the 1946 NSHD at age 7 years). This was necessary because a multilevel logistic regression model may not converge and, if it does converge, is likely to produce unstable estimates when one of the responses (e.g., obesity) includes only a few cases. Thinness could not be considered as a separate group for the same reason, and was recoded as missing because it is a risk factor for health outcomes and thus should not be combined with the normal weight group [46]. The referent group was, therefore, normal weight. The age scale was centred about the mean, and the shape of the trajectory was specified as a restricted cubic spline [47], with knots at equally spaced percentiles of the age distribution [48]. Models were tested with increasingly greater number of knots, with a minimum of three and a maximum equal to the number of sweeps minus one. The best model was selected based on a balance between the lowest BIC, the smallest differences between estimated probabilities and observed prevalence rates (divided by 100) of overweight or obesity, and a trajectory that provided the right degree of smoothing. In some instances it was necessary to remove random effects for the level two parameters (with little between-person variation) to achieve model convergence; no constraints were applied to the random effects variance-covariance matrix. The models were fitted using iterative generalized least squares and first order marginal quasi-likelihood. Trajectories and their 95% confidence intervals (CI) for each study were produced with probability of overweight or obesity on the *y*-axis and age on the *x*-axis in sex-specific plots.

The only age when all cohorts had sweeps was at 10 or 11 years, and additional analyses focusing on these data were conducted. Weight, height, and BMI were converted to sex- and age-specific Z-scores according to the UK—World Health Organisation (WHO) chart [49], and cohort-stratified box plots were produced. Because the Z-scores were skewed, Kruskal-Wallis tests with Bonferroni correction were used to test between-study differences.

LMSgrowth was used to compute childhood weight status and Z-scores and LMSchartmaker was used to fit the LMS models (http://www.healthforallchildren.com/). The Stata command *runmlwin*, which calls on MLwiN for model fitting, was used for the multilevel models [50]. All other analyses were performed in Stata 13 (StataCorp LP: College Station, TX, US).

## Results

In total, there were 273,843 BMI observations on 56,632 participants in studies spanning births between 1946–2001 and ages from 2–64 years (Table 1). Median BMI and the prevalence of overweight and obesity generally increased with age after mid-childhood in each study.

### Shifts in the Distribution of BMI

**Fig 1:**
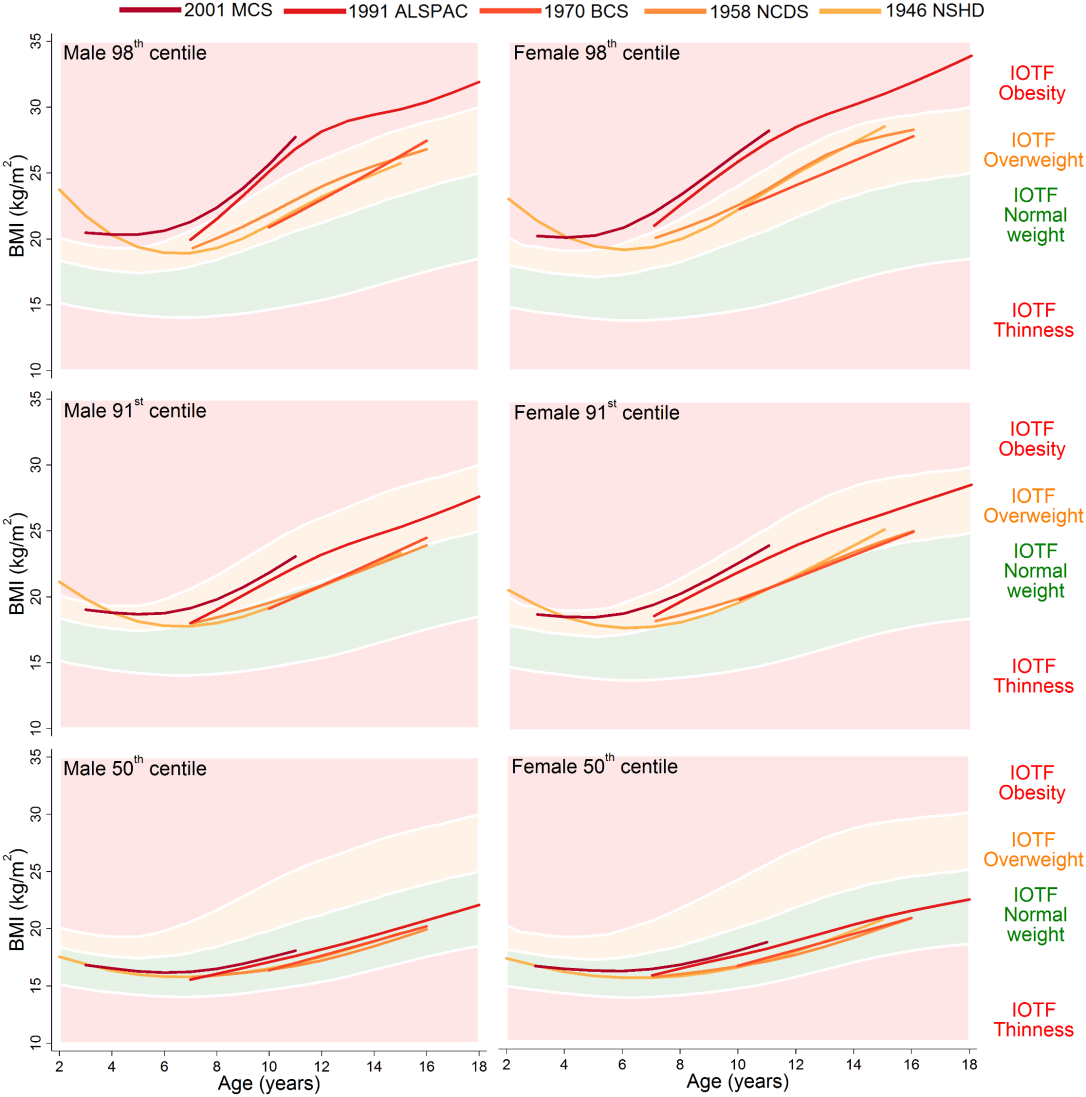
The 98th, 91st, and 50th childhood BMI centiles from sex- and study-stratified LMS models plotted against the IOTF cut-offs. BMI: Body Mass Index, IOTF: International Obesity Task Force, LMS: Lambda-Mu-Sigma, NSHD: Medical Research Council National Survey of Health and Development, NCDS National Child Development Study, BCS: British Cohort Study, ALSPAC: Avon Longitudinal Study of Parents and Children, MCS: Millennium Cohort Study.

**Fig 2:**
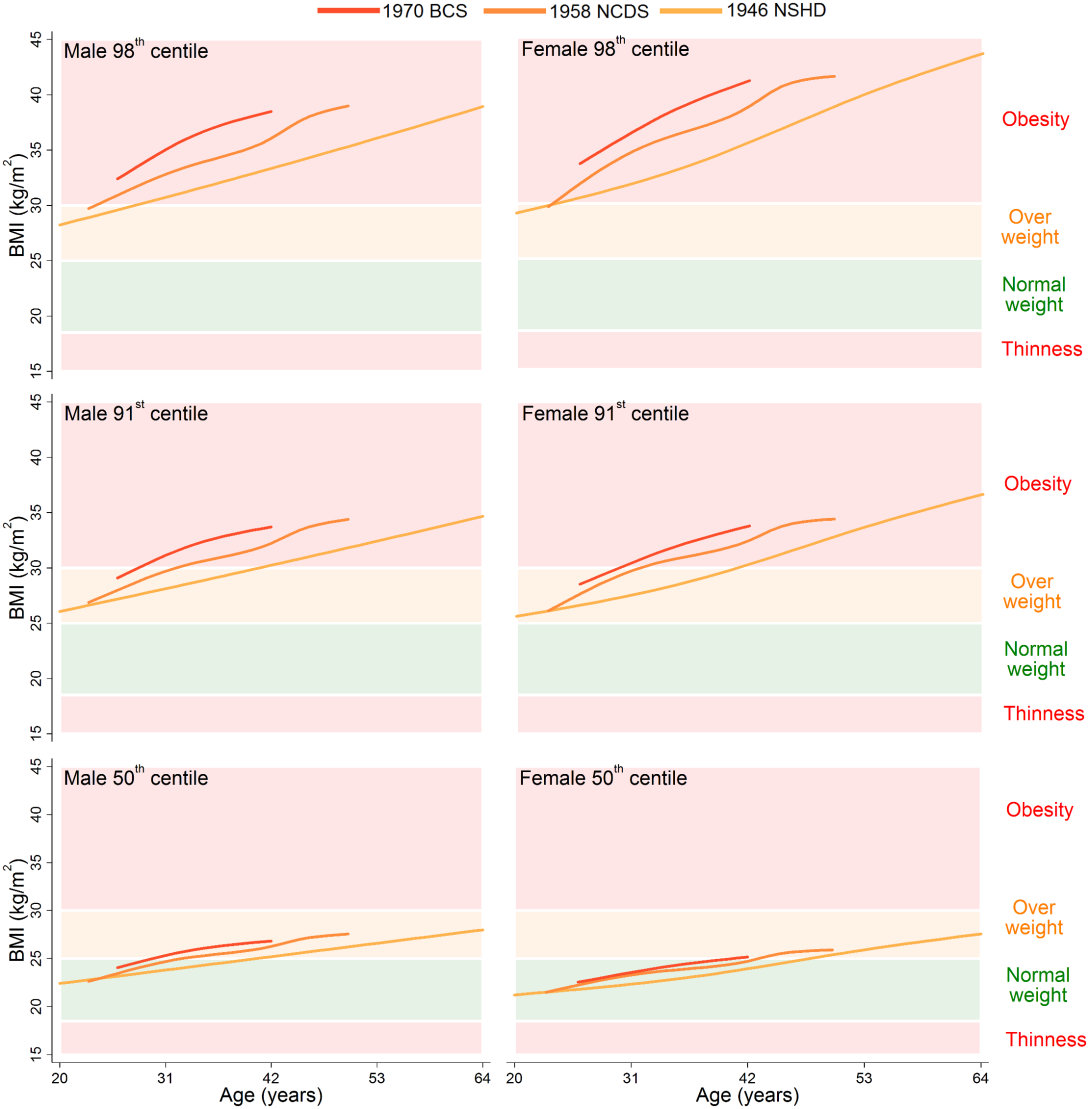
The 98^th^, 91^st^, and 50^th^ adulthood BMI centiles from sex- and study-stratified LMS models plotted against the normal cut-offs. BMI: Body Mass Index, LMS: Lambda-Mu-Sigma, NSHD: Medical Research Council National Survey of Health and Development, NCDS National Child Development Study, BCS: British Cohort Study.

The 98th, 91st, and 50th centiles are shown for childhood (ages 2–18 years) in Fig 1 and adulthood (ages 20–64 years) in Fig 2; model estimates and diagnostics can be found in S3–S7 Tables. During childhood, the 50th centiles for all studies lay in the middle of the normal weight range. There was little distinction in each of the centiles between the 1946 NSHD, 1958 NCDS, and 1970 BCS at overlapping ages, but large upward shifts at the 98th and 91st centiles and small upward shifts at the 50th centile were apparent for the 1991 ALSPAC and 2001 MCS. The exception to this pattern is the suggestive evidence that before 4 years of age, the 98th and 91st centiles were actually highest in the 1946 NSHD. During adulthood, separation between the 1946 NSHD, 1958 NCDS, and 1970 BCS centiles occurred, again with the greatest differences observed at the upper end of the BMI distribution. The age when a 50th centile first entered the overweight range was computed to the nearest year, and across the 1946 NSHD, 1958 NCDS, and 1970 BCS this age decreased from 41 to 33 to 30 years in males and 48 to 44 to 41 years in females. During childhood and adulthood, between-study differences in the 9th and 2nd centiles were negligible (S2 Fig and S3 Fig).

### Shifts in the Development of Overweight or Obesity

**Fig 3:**
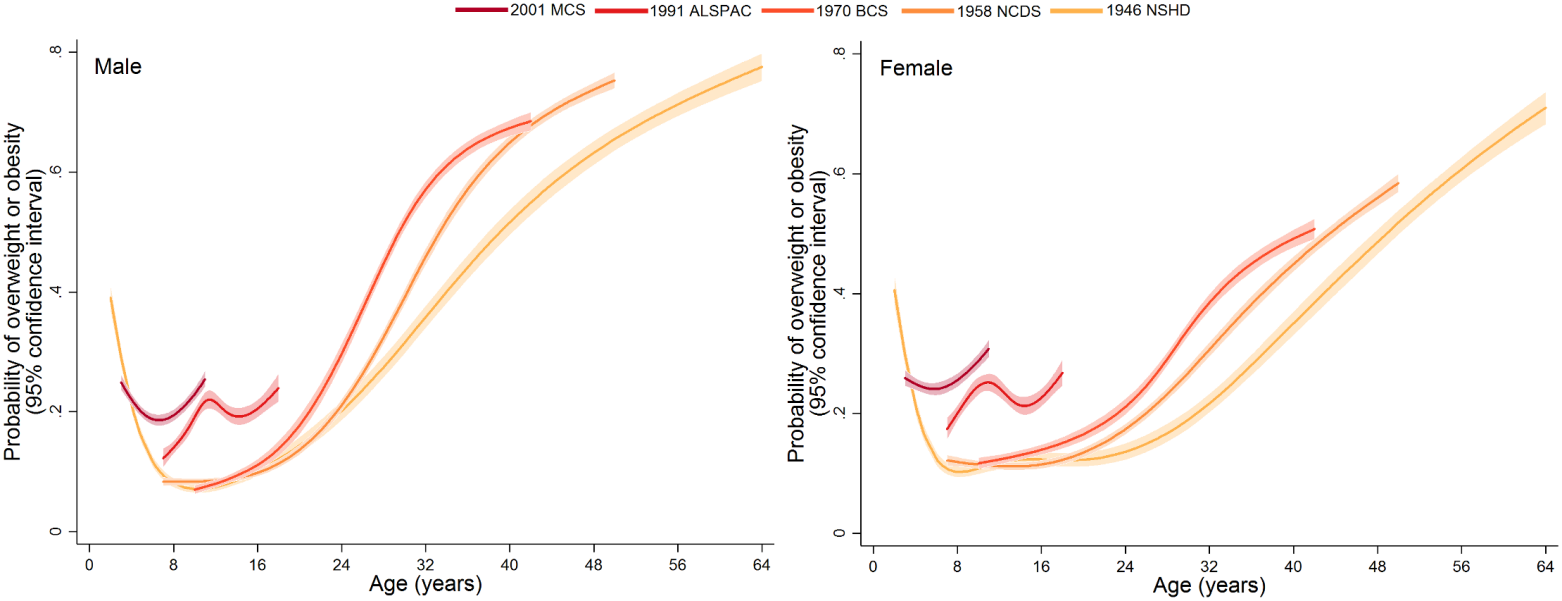
Trajectories of the probability of overweight or obesity (versus normal weight) from sex- and study-stratified multilevel logistic regression models. NSHD: Medical Research Council National Survey of Health and Development, NCDS National Child Development Study, BCS: British Cohort Study, ALSPAC: Avon Longitudinal Study of Parents and Children, MCS: Millennium Cohort Study.

**Fig 4:**
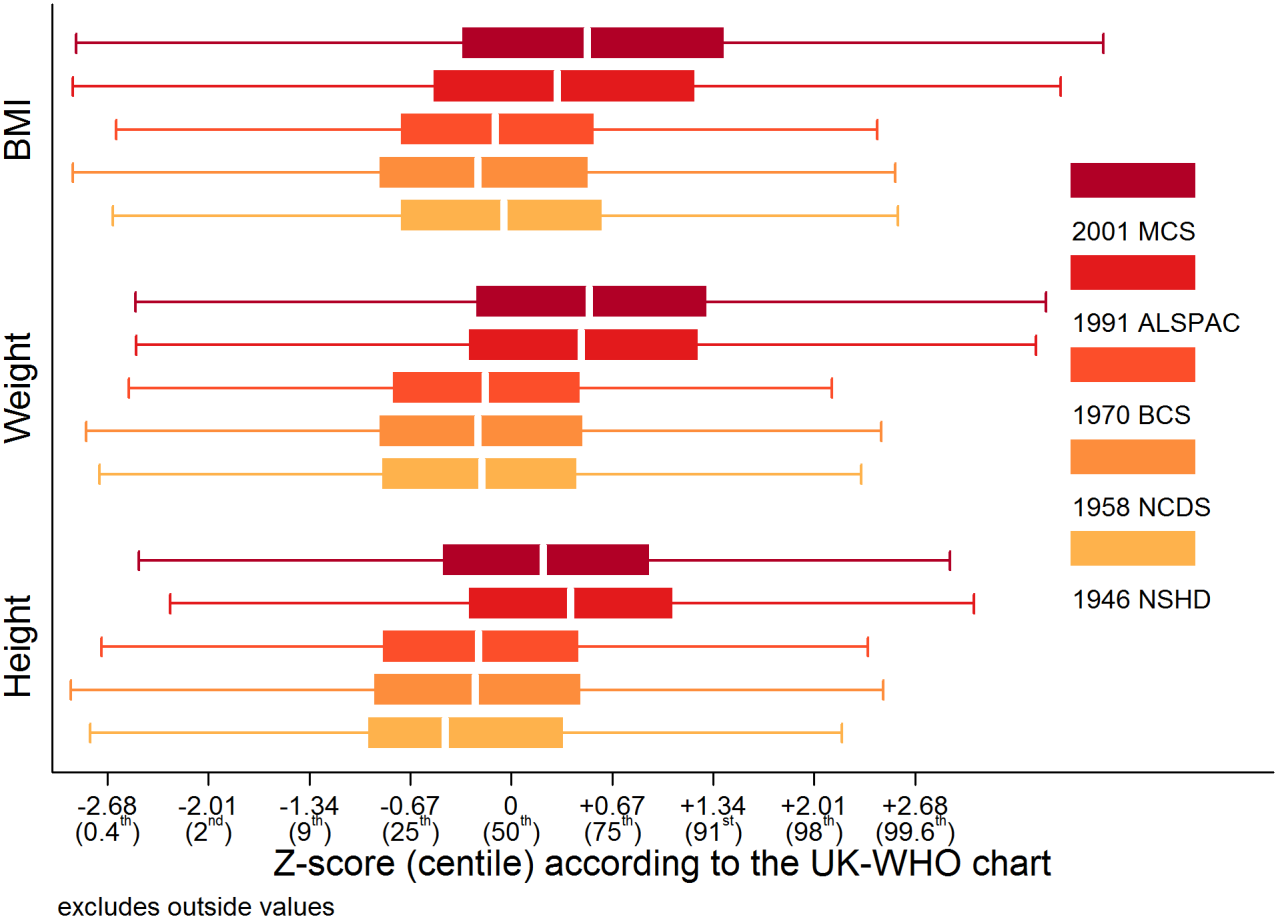
Study-stratified box plots for height, weight, and BMI Z-scores according to the UK-WHO chart at 10 or 11 years of age. BMI: Body Mass Index, UK-WHO: United Kingdom-World Health Organisation, NSHD: Medical Research Council National Survey of Health and Development, NCDS National Child Development Study, BCS: British Cohort Study, ALSPAC: Avon Longitudinal Study of Parents and Children, MCS: Millennium Cohort Study.

Trajectories describing the probability (or multiplied by 100 to get prevalence) of overweight or obesity are shown in Fig 3; model estimates and diagnostics etc. can be found in S8 Table and S9 Table. In the 1946 NSHD, 1958 NCDS, and 1970 BCS, the trajectories were initially similar in childhood at overlapping ages, after which they became steeper and diverged from one another. The probability of overweight or obesity in the 1958 NCDS diverged upward from that in the 1946 NSHD in early adulthood, as indicated by non-overlapping 95% CI (e.g., males at age 27 years: 0.294 [95% CI 0.283, 0.305] in 1958 NCDS; 0.255 [0.238, 0.273] in 1946 NSHD). However, the probability of overweight or obesity in the 1970 BCS diverged upward from that in the 1958 NCDS and 1946 NSHD in late adolescence (e.g., males at age 18 years: 0.138 [0.126, 0.151] in 1970 BCS; 0.113 [0.107, 0.121] in 1958 NCDS). Table 2 provides estimated probabilities at every decade of life in each study; even by 10 years of age, the probabilities in the 1991 ALSPAC and 2001 MCS were between two to three times those in the three older studies (e.g., males: 0.229 [0.219, 0.240] in 2001 MCS; 0.071 [0.065, 0.078] in 1946 NSHD). As shown in Fig 4, box plots revealed that observed median weight and height Z-scores at 10 or 11 years of age, as well as BMI Z-scores, were greater in the 1991 ALSPAC and 2001 MCS compared to the older three cohorts; these comparisons were nominally significant at *p* < 0.05 (S10 Table). Before age 4 years, the trajectories in Fig 3 suggest that children in the 1946 NSHD actually had the highest risks of overweight or obesity. Sensitivity analyses shown in S4 Fig found similar trajectories for participants with BMI data at all ages.

**Table 2:** Estimated probabilities of overweight or obesity (versus normal weight) from sex- and study-stratified multilevel logistic regression models.

		Male	Female
	Age	Estimate (95% Confidence Interval)	
**1946 NSHD**	10	0.071 (0.065, 0.078)	0.108 (0.099, 0.119)
	20	0.144 (0.131, 0.158)	0.123 (0.111, 0.135)
	30	0.316 (0.298, 0.334)	0.190 (0.175, 0.206)
	40	0.516 (0.495, 0.537)	0.350 (0.330, 0.370)
	50	0.655 (0.636, 0.674)	0.519 (0.498, 0.539)
	60	0.746 (0.724, 0.766)	0.661 (0.638, 0.684)
**1958 NCDS**	10	0.083 (0.078, 0.089)	0.115 (0.109, 0.121)
	20	0.137 (0.129, 0.145)	0.135 (0.127, 0.143)
	30	0.392 (0.379, 0.405)	0.269 (0.258, 0.280)
	40	0.649 (0.638, 0.661)	0.449 (0.437, 0.462)
	50	0.753 (0.740, 0.766)	0.585 (0.569, 0.600)
**1970 BCS**	10	0.070 (0.063, 0.078)	0.117 (0.107, 0.126)
	20	0.177 (0.163, 0.193)	0.165 (0.154, 0.177)
	30	0.517 (0.503, 0.530)	0.341 (0.329, 0.353)
	40	0.674 (0.660, 0.687)	0.492 (0.477, 0.506)
**1991 ALSPAC**	10	0.191 (0.178, 0.205)	0.245 (0.231, 0.261)
**2001 MCS**	10	0.229 (0.219, 0.240)	0.288 (0.276, 0.300)

NSHD: Medical Research Council National Survey of Health and Development, NCDS National Child Development Study, BCS: British Cohort Study, ALSPAC: Avon Longitudinal Study of Parents and Children, MCS: Millennium Cohort Study.

## Discussion

This study utilised the extensive longitudinal BMI data in the four national representative UK birth cohort studies, plus the 1991 ALSPAC, to investigate shifts over time in the BMI distribution and trajectories of overweight or obesity in a high-income country. The key findings were that (1) between-study differences in BMI occurred at the upper end of the distribution; (2) the median child in each study was normal weight, but the median adult was first overweight at increasingly younger ages; (3) cohorts born before the 1980s demonstrated increases in the probability of overweight or obesity across adulthood, with cohorts born more recently showing rapid increases at earlier ages; and (4) cohorts born after the 1980s already had probabilities of overweight or obesity in childhood that were two to three times greater than those for cohorts born before the 1980s. The childhood data may be reassuring in the sense that BMI values have remained relatively stable over time and within the normal range for the majority of the population. The UK is still, however, in a situation in which many more children are overweight or obese than in previous generations and, if the observed trends in adulthood BMI continue, the majority of children are likely to develop overweight or obesity at some point in their lives and at younger ages than did previous generations.

The main strength of the paper is the thorough analysis of 273,843 BMI observations on 56,632 participants in studies spanning births between 1946–2001 and ages from 2–64 years. No other study has such extensive serial data covering such a wide range of ages and birth years. In terms of weaknesses, (1) it was not possible to model separate trajectories for overweight and obesity; (2) the trajectories were smoothed over age periods in which no sweep took place and thus did not capture local traits, such as a peak during puberty, for some studies; (3) we assume our findings are due to changes in adiposity more so than fat-free mass, but this might not always be the case [51,52]; and (4) by excluding non-white participants, we were not able to consider the extent to which secular trends in obesity might be driven by the changing ethnic composition of the UK. This topic is perhaps more relevant to studies of more recent secular trends, at times when the ethnic composition of the UK has been less stable. Data from the HSE between 1998–2009 suggest that secular trends in overweight or obesity in ethnic minority groups do not follow those of white English children (e.g., obesity rates appear to have plateaued in white English children, but are continuing to rise in black Caribbean children) [53], thereby suggesting that our results are unlikely to be generalizable to other ethnic groups. The measurement protocols for weight and height were not consistent within and between-studies, which could have introduced bias if, for example, self-reported measurements were systemically under or over-reported. The tendency of people with greater BMIs to under-report weight suggests that our results are conservative, if anything [54,55]. The multilevel models were not conditional on any other covariates and therefore made the assumption that trajectories for individuals with missing BMI observations were similar to those whose BMI was observed at all ages. Sensitivity analyses found similar trajectories (compared to those reported in the paper) for participants with BMI data at all ages, thereby providing no evidence to suggest that (1) BMI was not missing at random or (2) there was a “healthy survivor effect” (i.e., those with healthy BMI values were surviving longer and contributing more data at older ages). We cannot be certain that our results are representative of secular trends in obesity development in other settings, although we imagine that similar processes have occurred in most high-income countries.

The observed prevalence rates of overweight or obesity in the 1946 NSHD, 1958 NCDS, and 1970 BCS at the most recent sweeps approximately matched those reported in the 2012 HSE at comparable adulthood ages [2], and the prevalence rates in the 2001 MCS approximately matched those reported in the 2012–2013 NCMP at age 11 years [7]. This suggests that our results are representative of the current epidemic at the national level. While the cross-sectional surveys have been able to stratify results by different divisions of society, such as ethnic group, the present paper was able to describe the age-related processes that have led to the current obesity epidemic. This work builds on the publication of Li et al. that investigated mean BMI trajectories in the 1946 NSHD and 1958 NCDS [21]. Both studies provide evidence that changes in the environment, largely starting in the 1980s, were responsible for increased risk of overweight or obesity mostly starting at 20–30 years of age. By the inclusion of more recently born cohorts, the present paper further demonstrates how children born after the 1980s have increased risk of overweight or obesity (compared to those born before the 1980s) because of earlier exposure to the obesogenic environment. Outside of the UK, this work adds to the large body of literature from studies in diverse settings around the world reporting secular trends toward positive skewing of the BMI distribution at cross-sectional ages [28–33] and the much smaller body of literature, mainly in the US, reporting secular trends in mean BMI trajectories [25–27].

There are two findings that warrant brief discussion in the context of public health implications. The first, that children in the 1946 NSHD appeared to have the highest risks of overweight or obesity before age 4 years, is in agreement with (1) other secular trend studies showing a decline in maximum infant BMI over time and (2) epidemiological studies showing that low rather than high infant BMI is deleterious in older cohorts [27,56,57]. The second, that at 10 or 11 years of age modern-day children appeared to be taller as well as heavier than children in previous generation, is in agreement with literature showing obese children to be temporarily taller than their non-obese peers at pubertal ages due to an advanced pace of development [58–60]. Clinical assessment of overweight or obesity risk in puberty might, therefore, consider height as well as BMI.

Since the inception of the obesity epidemic, the UK has not matched gains made in childhood and young adulthood mortality by comparable European Union member states [61]. Our results suggest that more recently born people accumulate greater exposure to overweight or obesity across their lives, and there is a large body of literature linking such “accumulation of risk” to a wide range of health outcomes, including CHD, type 2 diabetes, hypertension, and osteoarthritis [14–17,62]. Understanding the characteristics of individuals who maintain a normal BMI throughout their lives may also help with designing novel interventions and clinical recommendations on weight maintenance. Currently, there is a dearth of research and trials in this area [63]. It is obviously important to intervene early to prevent overweight or obesity, but there is also evidence to suggest that losing weight at any age in early to mid-adulthood is beneficial, for example to vascular health [64]. Despite the importance of life-course adiposity in disease processes being well-documented, future research is warranted to investigate the extent to which the documented shifts in the BMI distribution and overweight or obesity trajectories, which have resulted in greater accumulation of risk, have contributed to changes over time in adiposity-related morbidity and mortality. The plateauing of adulthood type 2 diabetes rates in the US between 2008–2012 might reflect a lagged effect of there being no change in the prevalence of obesity since 2003–2004, for example [65,66]. Changes in obesity prevalence in low- and middle-income countries may impact differently on rates on adiposity-related diseases than those in high-income countries because of contextual differences like access to health care and medication. Indeed, the availability of medication in high-income countries is one explanation for why CHD rates have declined in most countries over the past 30 years, in spite of adulthood obesity rates continuing to rise [67]. Nevertheless, being overweight or obese is bad for health at the individual level. Investigation of which changes over time in the environment have caused the secular trends documented in the present paper is also needed to further knowledge on the aetiology of overweight or obesity. In the absence of a series of national birth cohort studies, comparable work in other settings may be possible through exploitation of routine anthropometric data and record linkage, as demonstrated by publications using the Copenhagen School Health Records Register [68,69].

In conclusion, our results demonstrate how a population in a high-income country is experiencing greater risk of overweight or obesity due to secular trends at increasingly younger ages toward (1) positive skewing of the BMI distribution and (2) life-course overweight or obesity trajectories that are generally steeper and higher (i.e., more deleterious). If the secular trends persist, then modern-day and successive generations of children will accumulate greater overweight or obesity exposure across their lives than previous generations. Given our knowledge that such accumulation of exposure increases risk for diseases like CHD and type 2 diabetes, overweight and obesity will have severe public health consequences in decades to come, in the absence of effective intervention.

## Supporting Information

S1 Checklist STROBE checklist. (DOC)

S1 Fig Sample selection in the five UK birth cohort studies. BMI: Body Mass Index, UK: United Kingdom, NSHD: Medical Research Council National Survey of Health and Development, NCDS: National Child Development Study, BCS: British Cohort Study, ALSPAC: Avon Longitudinal Study of Parents and Children, MCS: Millennium Cohort Study.
(TIF)

S2 Fig The 9th and 2nd childhood BMI centiles from sex- and study-stratified LMS models plotted against the IOTF cut-offs. BMI: Body Mass Index, IOTF: International Obesity Task Force, LMS: Lambda-Mu-Sigma, NSHD: Medical Research Council National Survey of Health and Development, NCDS National Child Development Study, BCS: British Cohort Study, ALSPAC: Avon Longitudinal Study of Parents and Children, MCS: Millennium Cohort Study.
(TIF)

S3 Fig The 9th and 2nd adulthood BMI centiles from sex- and study-stratified LMS models plotted against the normal cut-offs. BMI: Body Mass Index, LMS: Lambda-Mu-Sigma, NSHD: Medical Research Council National Survey of Health and Development, NCDS National Child Development Study, BCS: British Cohort Study.
(TIF)

S4 Fig Trajectories of the probability of overweight or obesity (versus normal weight) from sex- and study-stratified multilevel logistic regression models applied in sensitivity analyses to participants with BMI data at all ages. NSHD: Medical Research Council National Survey of Health and Development (225 males and 244 females), NCDS National Child Development Study (1,145 males and 1,100 females), BCS: British Cohort Study (547 males and 1,106 females), ALSPAC: Avon Longitudinal Study of Parents and Children (912 males and 1,028 females), MCS: Millennium Cohort Study (3,499 males and 3,495 females).
(TIF)

S1 Table Main differences in measurement protocols for the weight and height data used in this paper. (DOCX)

S2 Table Main steps used to make the weight and height data from the different studies as comparable as possible in this paper. (DOCX)

S3 Table Childhood LMS values at target assessment ages from sex- and study-stratified models applied to serial BMI data. (DOCX)

S4 Table Adulthood LMS values at target assessment ages from sex- and study-stratified models applied to serial BMI data. (DOCX)

S5 Table Percentages of childhood BMI values above select centiles, estimated from sex- and study-stratified LMS models. (DOCX)

S6 Table Percentages of adulthood BMI values above select centiles, estimated from sex- and study-stratified LMS models. (DOCX)

S7 Table Age scale and EDFs used in the fitting of sex-, study-, and life-course-stage—stratified LMS models applied to serial BMI data. (DOCX)

S8 Table Study-stratified, binary logistic, multilevel models in males to describe overweight or obesity (versus normal weight) as a restricted cubic spline of age. (DOCX)

S9 Table Study-stratified, binary logistic, multilevel models in females to describe overweight or obesity (versus normal weight) as a restricted cubic spline of age. (DOCX)

S10 Table Kruskal-Wallis tests of between-study differences in height, weight, and BMI Z-scores according to the UK-WHO chart at 10 or 11 years of age; diagonal values are medians (interquartile range), and off-diagonal values are Bonferroni-corrected *p*-values. (DOCX)

## Abbreviations

ALSPAC: Avon Longitudinal Study of Parents and Children
BIC: Bayesian Information Criterion
BCS: British Cohort Study
BMI: body mass index
CHD: Coronary Heart Disease
CI: confidence interval
EDF: Equivalent degrees of freedom
HSE: Health Survey for England
IOTF: International Obesity Task Force
LMS: Lambda-Mu-Sigma
MCS: Millennium Cohort Study
NCDS: National Child Development Study
NCMP: National Child Measurement Programme for England
NHANES: National Health and Nutrition Examination Survey
NHS: National Health Service
NSHD: National Survey of Health and Development
