# Supplementary material for: How Has the Age-Related Process of Overweight or Obesity Development Changed over Time? Co-ordinated Analyses of Individual Participant Data from Five United Kingdom Birth Cohorts
Source: PLoS Med. 2015 May 19;12(5):e1001828. doi: 10.1371/journal.pmed.1001828 (PMC4437909; doi:10.1371/journal.pmed.1001828)
Supplement: S1 Checklist — (DOC) [file pmed.1001828.s001.doc]

STROBE Statement—Checklist of items that should be included in reports of ***cohort studies***

|  | Item No | Recommendation |
| --- | --- | --- |
| **Title and abstract** | 1 | (*a*) Indicate the study’s design with a commonly used term in the title or the abstract  Page 1 “How has the age-related process of overweight or obesity development changed over time? Co-ordinated analyses of individual participant data from five United Kingdom birth cohorts”. |
|  |  | (*b*) Provide in the abstract an informative and balanced summary of what was done and what was found  Page 2-3 (see methods and findings section). |
| Introduction | | |
| Background/rationale | 2 | Explain the scientific background and rationale for the investigation being reported  Page 4-6 (see introduction). |
| Objectives | 3 | State specific objectives, including any prespecified hypotheses  Page 6 “The present study utilises the extensive longitudinal data on BMI in the UK birth cohort studies, with the aims to investigate 1) shifts over time in the distribution of BMI across age and 2) shifts over time in the development of overweight or obesity across age.” |
| Methods | | |
| Study design | 4 | Present key elements of study design early in the paper  Page 1 “How has the age-related process of overweight or obesity development changed over time? Co-ordinated analyses of individual participant data from five United Kingdom birth cohorts”. |
| Setting | 5 | Describe the setting, locations, and relevant dates, including periods of recruitment, exposure, follow-up, and data collection  Page 6-7 (see study samples). |
| Participants | 6 | (*a*) Give the eligibility criteria, and the sources and methods of selection of participants. Describe methods of follow-up  Page 7 “The inclusion criteria for the present study excluded groups likely to have particularly different BMI values, thereby ensuring each study sample comprised a comparable composition of people. The criteria were 1) part of the original cohort (i.e., not an immigrant), 2) white race, 3) singleton birth, and 4) survival to at least age nine months in the 2001 MCS; age one year in the 1946 NSHD, 1958 NCDS, and 1991 ALSPAC; and age five years in the 1970 BCS. Each study determined race/ ethnicity differently and not always consistently across all sweeps; we use “White” as it best captures everyone in our sample (i.e., everyone was assumed to be White British in the 1946 NSHD, but in the 1958 NCDS, 1970 BCS, 1991 ALSPAC, and 2001 MCS the samples were largely restricted to the responses “European/Caucasian”, “European/UK”, “White”, and “White”, respectively). Participants were not excluded on the basis of missing data for these variables, but were required to have sex recorded and at least one observation of BMI. Sample selection is shown in S1 Fig and sample sizes are shown in Table 1”. |
|  |  | (*b*) For matched studies, give matching criteria and number of exposed and unexposed  N/A. |
| Variables | 7 | Clearly define all outcomes, exposures, predictors, potential confounders, and effect modifiers. Give diagnostic criteria, if applicable  Page 10 “BMI was computed as weight (kg)/ height (m)^2^ and thinness, overweight, and obesity were defined according to International Obesity Task Force (IOTF) cut-offs during childhood and standard cut-offs of 18.5, 25, and 30 kg/m^2^ during adulthood. The IOTF cut-offs are centiles spanning 2-18 years of age that pass through the adulthood cut-offs at age 18 years (e.g., approximately the 90^th^ IOTF centile is used to define overweight in boys as it passes through the adulthood cut-off of 25 kg/m^2^ at age 18 years), thereby avoiding artificial change in prevalence during the transition to adulthood.” |
| Data sources/ measurement | 8* | For each variable of interest, give sources of data and details of methods of assessment (measurement). Describe comparability of assessment methods if there is more than one group  Page 10 “S1 Table summarises the main differences in measurement protocols and S2 Table describes the main steps used to make the anthropometry as comparable as possible”. |
| Bias | 9 | Describe any efforts to address potential sources of bias  Page 10 “S1 Table summarises the main differences in measurement protocols and S2 Table describes the main steps used to make the anthropometry as comparable as possible”.  Page 15 “Sensitivity analyses shown in S4 Fig found similar trajectories for participants with BMI data at all ages.” |
| Study size | 10 | Explain how the study size was arrived at  Page 7 “The inclusion criteria for the present study excluded groups likely to have particularly different BMI values, thereby ensuring each study sample comprised a comparable composition of people. The criteria were 1) part of the original cohort (i.e., not an immigrant), 2) white race, 3) singleton birth, and 4) survival to at least age nine months in the 2001 MCS; age one year in the 1946 NSHD, 1958 NCDS, and 1991 ALSPAC; and age five years in the 1970 BCS. Each study determined race/ ethnicity differently and not always consistently across all sweeps; we use “White” as it best captures everyone in our sample (i.e., everyone was assumed to be White British in the 1946 NSHD, but in the 1958 NCDS, 1970 BCS, 1991 ALSPAC, and 2001 MCS the samples were largely restricted to the responses “European/Caucasian”, “European/UK”, “White”, and “White”, respectively). Participants were not excluded on the basis of missing data for these variables, but were required to have sex recorded and at least one observation of BMI. Sample selection is shown in S1 Fig and sample sizes are shown in Table 1” |
| Quantitative variables | 11 | Explain how quantitative variables were handled in the analyses. If applicable, describe which groupings were chosen and why  Page 10 “BMI was computed as weight (kg)/ height (m)^2^ and thinness, overweight, and obesity were defined according to International Obesity Task Force (IOTF) cut-offs during childhood and standard cut-offs of 18.5, 25, and 30 kg/m^2^ during adulthood. The IOTF cut-offs are centiles spanning 2-18 years of age that pass through the adulthood cut-offs at age 18 years (e.g., approximately the 90^th^ IOTF centile is used to define overweight in boys as it passes through the adulthood cut-off of 25 kg/m^2^ at age 18 years), thereby avoiding artificial change in prevalence during the transition to adulthood.” |
| Statistical methods | 12 | (*a*) Describe all statistical methods, including those used to control for confounding  Page 10-13 (see statistical analysis). |
|  |  | (*b*) Describe any methods used to examine subgroups and interactions  Page 11. The Lambda-Mu-Sigma (LMS) method was used to summarise the distribution of BMI across age in sex, study, and childhood (ages 2-18 years) versus adulthood (ages 20-64 years) stratified models.  Page 11. Sex and study stratified binary logistic multilevel models (observations at level one nested within individuals at level two) were used to describe weight status trajectories, assuming missing data were at random. |
|  |  | (*c*) Explain how missing data were addressed  Page 11 “assuming missing data were at random.” |
|  |  | (*d*) If applicable, explain how loss to follow-up was addressed  Page 11 “assuming missing data were at random.” |
|  |  | (*e*) Describe any sensitivity analyses  N/A. |
| Results | | |
| Participants | 13* | (a) Report numbers of individuals at each stage of study—eg numbers potentially eligible, examined for eligibility, confirmed eligible, included in the study, completing follow-up, and analysed  Page 8 (table 1). |
|  |  | (b) Give reasons for non-participation at each stage  Page 11 “assuming missing data were at random.” |
|  |  | (c) Consider use of a flow diagram  Page 7 (S1 Fig). |
| Descriptive data | 14* | (a) Give characteristics of study participants (eg demographic, clinical, social) and information on exposures and potential confounders  Page 8 (table 1). |
|  |  | (b) Indicate number of participants with missing data for each variable of interest  Page 8 (table 1). |
|  |  | (c) Summarise follow-up time (eg, average and total amount)  Page 13 “In total, there were 273,843 BMI observations on 56,632 participants in studies spanning births between 1946-2001 and ages from 2-64 years (Table 1).” |
| Outcome data | 15* | Report numbers of outcome events or summary measures over time  Page 8 (table 1). |
| Main results | 16 | (*a*) Give unadjusted estimates and, if applicable, confounder-adjusted estimates and their precision (eg, 95% confidence interval). Make clear which confounders were adjusted for and why they were included  Page 13-15 (S3-10 Tables). |
|  |  | (*b*) Report category boundaries when continuous variables were categorized  Page 10 “BMI was computed as weight (kg)/ height (m)^2^ and thinness, overweight, and obesity were defined according to International Obesity Task Force (IOTF) cut-offs during childhood and standard cut-offs of 18.5, 25, and 30 kg/m^2^ during adulthood. The IOTF cut-offs are centiles spanning 2-18 years of age that pass through the adulthood cut-offs at age 18 years (e.g., approximately the 90^th^ IOTF centile is used to define overweight in boys as it passes through the adulthood cut-off of 25 kg/m^2^ at age 18 years), thereby avoiding artificial change in prevalence during the transition to adulthood.” |
|  |  | (*c*) If relevant, consider translating estimates of relative risk into absolute risk for a meaningful time period  N/A. |
| Other analyses | 17 | Report other analyses done—eg analyses of subgroups and interactions, and sensitivity analyses  Page 13-15 (results). |
| Discussion | | |
| Key results | 18 | Summarise key results with reference to study objectives  Page 16 “This study utilised the extensive longitudinal BMI data in the four national representative UK birth cohort studies, plus the 1991 ALSPAC, to investigate shifts over time in the BMI distribution and trajectories of overweight or obesity in a high-income country. The key findings were that 1) between-study differences in BMI occurred at the upper end of the distribution, 2) the median child in each study was normal weight but the median adult was first overweight at increasingly younger ages, 3) cohorts born before the 1980’s demonstrated increases in the probability of overweight or obesity across adulthood, with cohorts born more recently showing rapid increases at earlier ages, and 4) cohorts born after the 1980’s already had probabilities of overweight or obesity in childhood that were two to three times greater than those for cohorts born before the 1980’s.” |
| Limitations | 19 | Discuss limitations of the study, taking into account sources of potential bias or imprecision. Discuss both direction and magnitude of any potential bias  Page 16-17 “The main strength of the paper is the thorough analysis of 273,843 BMI observations on 56,632 participants in studies spanning births between 1946-2001 and ages from 0-64 years. No other study has such extensive serial data covering such a wide range of ages and birth years. In terms of weaknesses, 1) it was not possible to model separate trajectories for overweight and obesity; 2) the trajectories were smoothed over age periods in which no sweep took place and thus did not capture local traits, such as a peak during puberty, for some studies; 3) we assume our findings are due to changes in adiposity more so than fat-free mass, but this might not always be the case ; and 4) by excluding non-White participants, we were not able to consider the extent to which secular trends in obesity might be driven by the changing ethnic composition of the UK. This topic is perhaps more relevant to studies of more recent secular trends, at times when the ethnic composition of the UK has been less stable. Data from the HSE between 1998-2009 suggest that secular trends in overweight or obesity in ethnic minority groups do not follow those of white English children , thereby providing evidence that our results are not generalizable to other ethnic groups. The measurement protocols for weight and height were not consistent within and between-studies, which could have introduced bias if, for example, self-reported measurements were systemically under or over-reported. The tendency of people with greater BMI’s to under-report weight suggests that our results are conservative, if anything . The multilevel models were not conditional on any other covariates and therefore made the assumption that trajectories for individuals with missing BMI observations were similar to those whose BMI was observed at all ages. Sensitivity analyses found similar trajectories (compared to those reported in the paper) for participants with BMI data at all ages, thereby providing no evidence to suggest that 1) BMI was not missing at random or 2) there was a “healthy survivor effect” (i.e., those with healthy BMI values were surviving longer and contributing more data at older ages). We cannot be certain that our results are representative of secular trends in obesity development in other settings, although we imagine that similar processes have occurred in most high-income countries.” |
| Interpretation | 20 | Give a cautious overall interpretation of results considering objectives, limitations, multiplicity of analyses, results from similar studies, and other relevant evidence  Page 21 “In conclusion, our results demonstrate how a population in a high-income country is experiencing greater risk of overweight or obesity due to secular trends at increasingly younger ages toward 1) positive skewing of the BMI distribution and 2) life course overweight or obesity trajectories that are generally steeper and higher (i.e., more deleterious). If the secular trends persist, then modern day and successive generations of children will accumulate greater overweight or obesity exposure across their lives than previous generations. Given our knowledge that such accumulation of exposure increases risk for diseases like CHD and type two diabetes, overweight and obesity will have devastating public health consequences in decades to come, in the absence of effective intervention.” |
| Generalisability | 21 | Discuss the generalisability (external validity) of the study results  Page 17 “Data from the HSE between 1998-2009 suggest that secular trends in overweight or obesity in ethnic minority groups do not follow those of white English children , thereby providing evidence that our results are not generalizable to other ethnic groups.” “We cannot be certain that our results are representative of secular trends in obesity development in other settings, although we imagine that similar processes have occurred in most high-income countries.” |
| Other information | | |
| Funding | 22 | Give the source of funding and the role of the funders for the present study and, if applicable, for the original study on which the present article is based  Page 31 “This project is part of a collaborative research programme entitled CLOSER. This programme is funded by the ESRC grant reference: ES/K000357/1. Each of the studies has received their own funding to collect the data used in the present paper; this information is available from the study websites and/ or cohort profiles. The funders had no role in study design, data collection and analysis, decision to publish, or preparation of the manuscript.” |

*Give information separately for exposed and unexposed groups.

**Note:** An Explanation and Elaboration article discusses each checklist item and gives methodological background and published examples of transparent reporting. The STROBE checklist is best used in conjunction with this article (freely available on the Web sites of PLoS Medicine at http://www.plosmedicine.org/, Annals of Internal Medicine at http://www.annals.org/, and Epidemiology at http://www.epidem.com/). Information on the STROBE Initiative is available at http://www.strobe-statement.org.
