# Supplementary figures and images for: How Has the Age-Related Process of Overweight or Obesity Development Changed over Time? Co-ordinated Analyses of Individual Participant Data from Five United Kingdom Birth Cohorts
Source: PLoS Med. 2015 May 19;12(5):e1001828. doi: 10.1371/journal.pmed.1001828 (PMC4437909; doi:10.1371/journal.pmed.1001828)

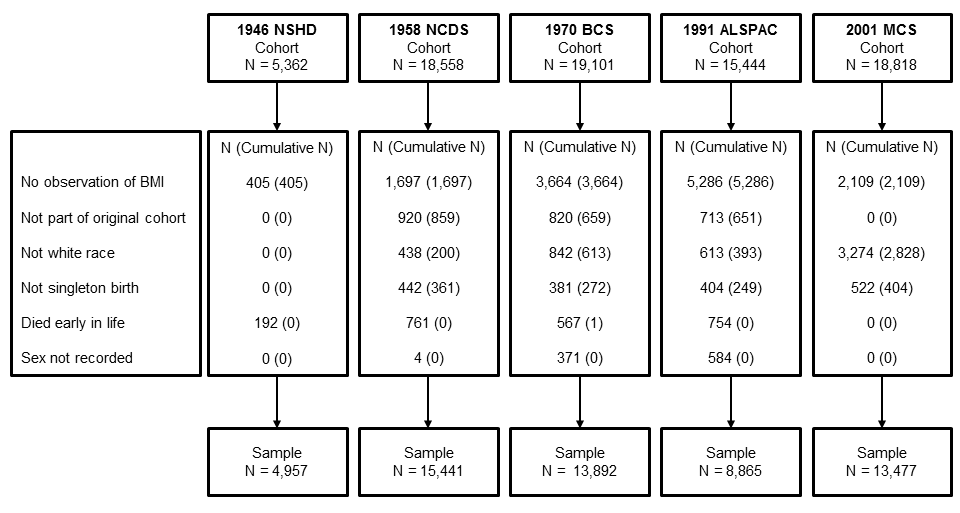

Supplement: S1 Fig — BMI: Body Mass Index, UK: United Kingdom, NSHD: Medical Research Council National Survey of Health and Development, NCDS: National Child Development Study, BCS: British Cohort Study, ALSPAC: Avon Longitudinal Study of Parents and Children, MCS: Millennium Cohort Study. (TIF) [file pmed.1001828.s002.tif]

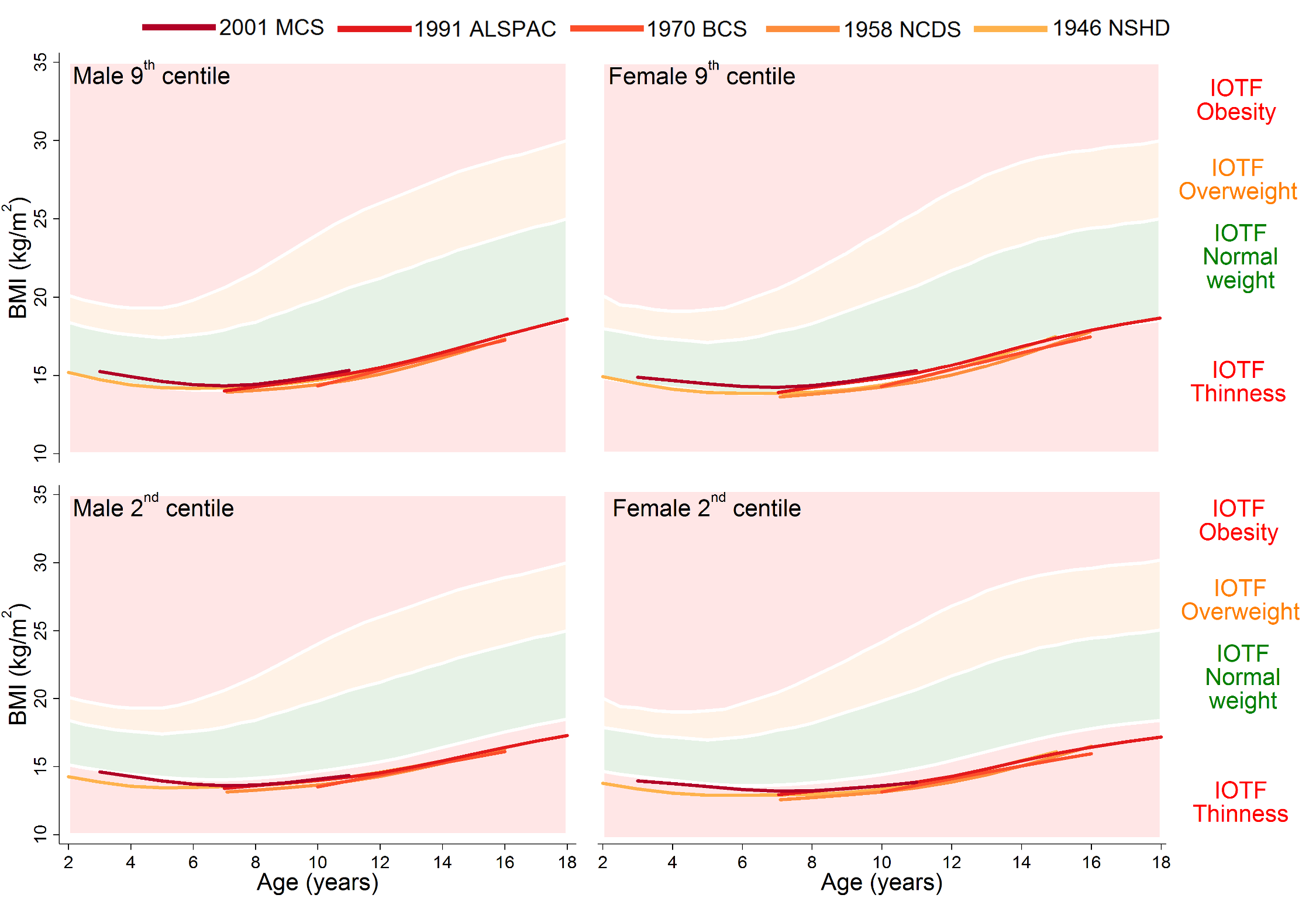

Supplement: S2 Fig — BMI: Body Mass Index, IOTF: International Obesity Task Force, LMS: Lambda-Mu-Sigma, NSHD: Medical Research Council National Survey of Health and Development, NCDS National Child Development Study, BCS: British Cohort Study, ALSPAC: Avon Longitudinal Study of Parents and Children, MCS: Millennium Cohort Study. (TIF) [file pmed.1001828.s003.tif]

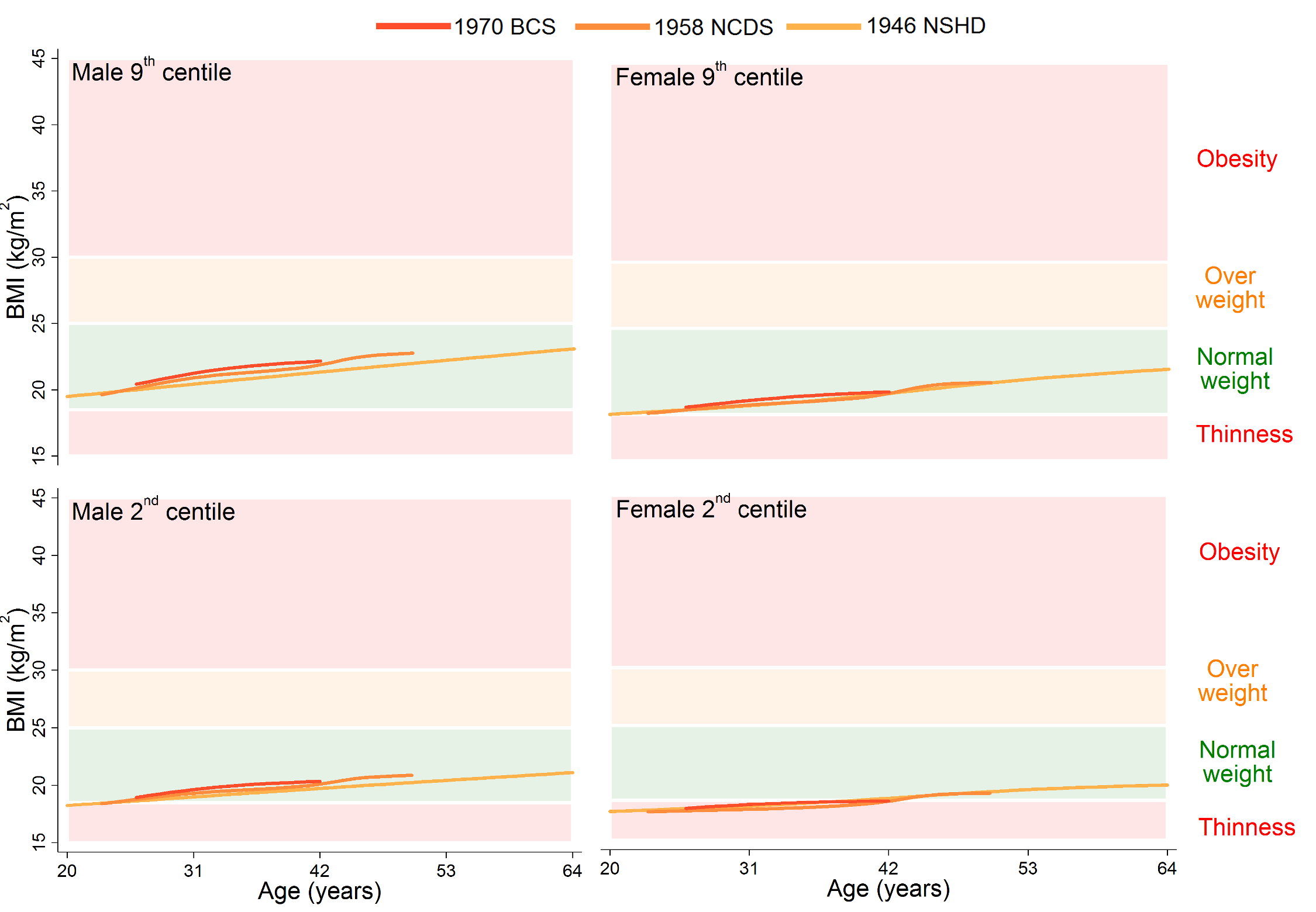

Supplement: S3 Fig — BMI: Body Mass Index, LMS: Lambda-Mu-Sigma, NSHD: Medical Research Council National Survey of Health and Development, NCDS National Child Development Study, BCS: British Cohort Study. (TIF) [file pmed.1001828.s004.tif]

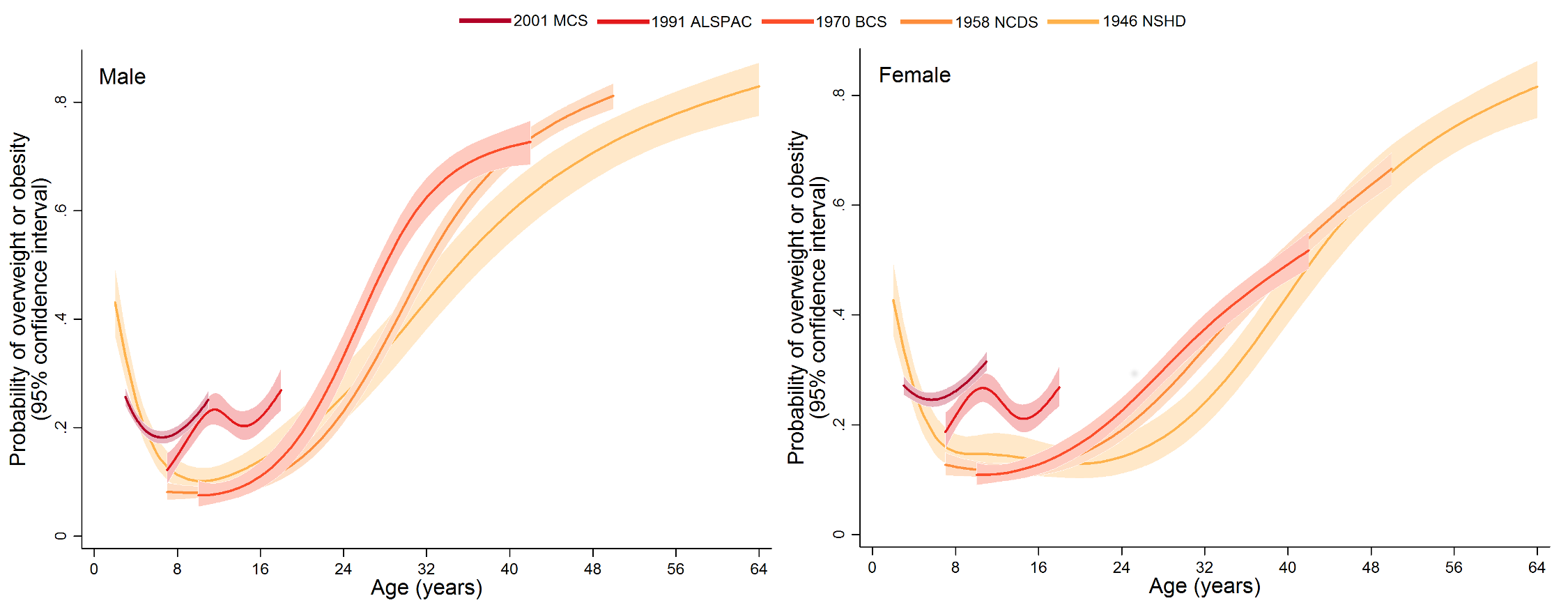

Supplement: S4 Fig — NSHD: Medical Research Council National Survey of Health and Development (225 males and 244 females), NCDS National Child Development Study (1,145 males and 1,100 females), BCS: British Cohort Study (547 males and 1,106 females), ALSPAC: Avon Longitudinal Study of Parents and Children (912 males and 1,028 females), MCS: Millennium Cohort Study (3,499 males and 3,495 females). (TIF) [file pmed.1001828.s005.tif]
