# Supplementary material for: How Has the Age-Related Process of Overweight or Obesity Development Changed over Time? Co-ordinated Analyses of Individual Participant Data from Five United Kingdom Birth Cohorts
Source: PLoS Med. 2015 May 19;12(5):e1001828. doi: 10.1371/journal.pmed.1001828 (PMC4437909; doi:10.1371/journal.pmed.1001828)
Supplement: S1 Table — (DOCX) [file pmed.1001828.s006.docx]

**S1 Table. Main differences in measurement protocols for the weight and height data used in this paper**

|  | Sweep  Target age (date) | Assessment type | System of  measurement | Precision of  weight measurement | Precision of  height measurement |
| --- | --- | --- | --- | --- | --- |
| **1946 NSHD** | 2 (1948) | Measured  (health visitor) | Imperial | 0.028 kg | 0.025 m |
|  | 4 (1950) | Measured  (health visitor) | Imperial | 0.113 kg | 0.025 m |
|  | 6 (1952) | Measured  (school doctor) | Imperial | 0.113 kg | 0.006 m |
|  | 7 (1953) | Measured  (school doctor) | Imperial | 0.028 kg | 0.006 m |
|  | 11 (1957) | Measured  (school doctor) | Imperial | 0.028 kg | 0.006 m |
|  | 15 (1961) | Measured  (school doctor) | Imperial | 0.028 kg | 0.006 m |
|  | 20 (1966) | Self-reported  (postal questionnaire) | Imperial | 0.454 kg | 0.025 m |
|  | 26 (1972) | Self-reported  (administered questionnaire) | Imperial | 0.454 kg | 0.025 m |
|  | 36 (1982) | Measured  (trained nurse) | Metric | 0.5 kg | 0.005 m |
|  | 43 (1989) | Measured  (trained nurse) | Metric | 0.5 kg | 0.001 m |
|  | 53 (1999) | Measured  (trained nurse) | Metric | 0.1 kg | 0.005 m |
|  | 60-64 (2006-2010) | Measured  (trained nurse) | Metric | 0.1 kg | 0.001 m |
| **1958 NCDS** | 7 (1965) | Measured  (medical officer) | Metric  or imperial | 0.454 kg | 0.01 m |
|  | 11 (1969) | Measured  (medical officer) | Imperial | 0.454 kg | 0.006 m |
|  | 16 (1974) | Measured  (medical officer) | Metric  or imperial | 0.01 to 0.454 kg | 0.006 to 0.01 m |
|  | 23 (1981) | Self-reported  (administered questionnaire) | Imperial | 0.454 kg | 0.025 m |
|  | 33 (1991) | Measured  (trained interviewer) | Metric | 0.1 kg | 0.01 m |
|  | 42 (2000) | Self-reported  (CAPI) | Metric  or imperial | 0.454 to 1 kg | 0.01 to 0.025 m |
|  | 44 (2002) | Measured  (trained nurse)  or self-reported  (CAPI) | Metric  or imperial | 0.1 to 0.454 kg | 0.001 m |
|  | 50 (2008) | Self-reported  (CAPI) | Metric  or imperial | 0.454 to 1 kg | 0.01 to 0.025 m |
| **1970 BCS** | 10 (1980) | Measured  (medical officer) | Metric  or imperial | 0.028 to 0.1 kg | 0.001 to 0.006 m |
|  | 16 (1986) | Measured  (medical officer)  or self-reported  (questionnaire) | Metric  or imperial | 0.028 to 0.1 kg | 0.005 to 0.006 m |
|  | 26 (1996) | Self-reported  (postal questionnaire) | Metric  or imperial | 0.454 to 1 kg | 0.01 to 0.025 m |
|  | 30 (2000) | Self-reported  (CAPI) | Metric  or imperial | 0.454 to 1 kg | 0.01 to 0.025 m |
|  | 34 (2004) | Self-reported  (CAPI) | Metric  or imperial | 0.454 to 1 kg | 0.01 to 0.025 m |
|  | 42 (2012) | Self-reported  (CAPI) | Metric  or imperial | 0.454 to 1 kg | 0.01 m |
| **1991 ALSPAC** | 7 (1998) | Measured  (anthropometrist) | Metric | 0.05 kg | 0.001 m |
|  | 8 (1999) | Measured  (anthropometrist) | Metric | 0.05 kg | 0.001 m |
|  | 9 (2000) | Measured  (anthropometrist) | Metric | 0.05 kg | 0.001 m |
|  | 10 (2001) | Measured  (anthropometrist) | Metric | 0.05 kg | 0.001 m |
|  | 11 (2002) | Measured  (anthropometrist) | Metric | 0.05 kg | 0.001 m |
|  | 13 (2004) | Measured  (anthropometrist) | Metric | 0.1 kg | 0.001 m |
|  | 14 (2005) | Measured  (anthropometrist) | Metric | 0.1 kg | 0.001 m |
|  | 15 (2006) | Measured  (anthropometrist) | Metric | 0.1 kg | 0.001 m |
|  | 18 (2009) | Measured  (anthropometrist) | Metric | 0.05 kg | 0.001 m |
| **2001 MCS** | 3 (2004) | Measured  (trained interviewer) | Metric | 0.001 kg | 0.001 m |
|  | 5 (2006) | Measured  (trained interviewer) | Metric | 0.1 kg | 0.001 m |
|  | 7 (2008) | Measured  (trained interviewer) | Metric | 0.1 kg | 0.001 m |
|  | 11 (2012) | Measured  (trained interviewer) | Metric | 0.1 kg | 0.001 m |

CAPI: Computer-Assisted Personal Interviewing, NSHD: Medical Research Council National Survey of Health and Development, NCDS National Child Development Study, BCS: British Cohort Study, ALSPAC: Avon Longitudinal Study of Parents and Children, MCS: Millennium Cohort Study
