# Supplementary material for: How Has the Age-Related Process of Overweight or Obesity Development Changed over Time? Co-ordinated Analyses of Individual Participant Data from Five United Kingdom Birth Cohorts
Source: PLoS Med. 2015 May 19;12(5):e1001828. doi: 10.1371/journal.pmed.1001828 (PMC4437909; doi:10.1371/journal.pmed.1001828)
Supplement: S2 Table — (DOCX) [file pmed.1001828.s007.docx]

**S2 Table. Main steps used to make the weight and height data from the different studies as comparable as possible in this paper**

| 1 | Weights and heights were converted to kilograms and metres, respectively, as necessary. |
| --- | --- |
| 2 | Measured data at age 16 years in the 1970 BCS were augmented with 2,353 self-reported weights and 2,309 self-reported heights at the same age to maximise the amount of available information. |
| 3 | Further, measured data at age 44 years in the 1958 NCDS were augmented with 12 observations of self-reported weight greater than 150 kg. This was done in an attempt to retrieve information from the upper end of the distribution that appeared to have been removed by the employment of a cut-off during data entry or cleaning. Similar situations were found for weight at age 7 years in the 1958 NCDS, and weight at age 10 years and height at age 26 years in the 1970 BCS, but in these instances it was not possible to retrieve any data. |
| 4 | Height was only reported at age 50 years in the 1958 NCDS if it had not been measured at the previous sweep at age 44 years; 9,063 missing observations of height at age 50 years were filled in with observations of height from the sweep at age 44 years. The same strategy had already been applied to study derived variables of height at ages 34 (filled in using age 30 year data) and 42 years (filled in using age 30 or 34 year data) in the 1970 BCS. |
| 5 | Where variables of decimal age at assessment were not available, they were computed from existing age variables or as the difference between date of birth and date of assessment. |
| 6 | For sweeps that were missing a date or some component of a date variable (i.e., day or month or year), day, month, and/ or year was assigned to the whole cohort; day was taken to be 15 in all studies, month to be 3 in the 1946 NSHD and 1958 NCDS and 4 in the 1970 BCS, and year to be that in which the sweep took place for the 1946 NSHD, 1958 NCDS, and 1970 BCS. |
| 7 | Participants who were still missing decimal age were assigned the mean value for that cohort at that sweep. |
| 8 | In some instances, no data on whether or not a woman was pregnant at a given sweep were recorded. Where it was possible to identify measurements taken while a woman was pregnant, these were excluded (1946 NSHD: 257 observations; 1958 NCDS: 684 observations; 1970 BCS: 110 observations). |
| 9 | A standardised data cleaning protocol was applied. This involved removal of biologically implausible values using sensible yet arbitrary cut-offs (e.g., weight > 250 kg and height > 3 m) and inspection of a connected scatter plot of serial weight or height against age (i.e., a trajectory) for persons with a measurement or change in measurement between two consecutive ages greater than five standard deviations from the sex and study stratified mean. The total number of weight observations excluded by this cleaning process in the 1946 NSHD, 1958 NCDS, 1970 BCS, 1991 ALSPAC, and 2001 MCS were 3, 371, 50, 10, and 90, respectively. For height, these numbers were 15, 296, 100, 24, and 16. |

NSHD: Medical Research Council National Survey of Health and Development, NCDS National Child Development Study, BCS: British Cohort Study, ALSPAC: Avon Longitudinal Study of Parents and Children, MCS: Millennium Cohort Study
