# Supplementary material for: How Has the Age-Related Process of Overweight or Obesity Development Changed over Time? Co-ordinated Analyses of Individual Participant Data from Five United Kingdom Birth Cohorts
Source: PLoS Med. 2015 May 19;12(5):e1001828. doi: 10.1371/journal.pmed.1001828 (PMC4437909; doi:10.1371/journal.pmed.1001828)
Supplement: S3 Table — (DOCX) [file pmed.1001828.s008.docx]

**S3 Table. Childhood LMS values at target assessment ages from sex and study stratified models applied to serial BMI data**

|  |  | **Male** | | | **Female** | | |
| --- | --- | --- | --- | --- | --- | --- | --- |
|  | Age | L | M | S | L | M | S |
| **1946 NSHD** | 2 | -1.494 | 17.554 | 0.122 | -1.331 | 17.242 | 0.120 |
|  | 4 | -0.716 | 16.351 | 0.100 | -0.822 | 16.064 | 0.103 |
|  | 6 | -0.668 | 15.810 | 0.085 | -1.098 | 15.554 | 0.094 |
|  | 7 | -0.917 | 15.795 | 0.083 | -1.397 | 15.547 | 0.095 |
|  | 11 | -1.912 | 16.972 | 0.105 | -1.828 | 17.073 | 0.124 |
|  | 15 | -0.985 | 19.646 | 0.119 | -1.124 | 20.617 | 0.136 |
| **1958 NCDS** | 7 | -0.659 | 15.737 | 0.096 | -1.108 | 15.593 | 0.110 |
|  | 11 | -2.161 | 16.759 | 0.114 | -1.629 | 16.977 | 0.131 |
|  | 16 | -1.588 | 19.931 | 0.118 | -1.294 | 20.745 | 0.128 |
| **1970 BCS** | 10 | -1.141 | 16.356 | 0.107 | -1.223 | 16.585 | 0.124 |
|  | 16 | -1.141 | 20.198 | 0.129 | -0.523 | 20.734 | 0.136 |
| **1991 ALSPAC** | 7 | -2.639 | 15.553 | 0.091 | -2.116 | 15.743 | 0.108 |
|  | 8 | -2.536 | 16.063 | 0.103 | -1.962 | 16.345 | 0.121 |
|  | 9 | -2.434 | 16.569 | 0.116 | -1.809 | 16.928 | 0.134 |
|  | 10 | -2.331 | 17.078 | 0.127 | -1.699 | 17.477 | 0.145 |
|  | 11 | -2.224 | 17.614 | 0.137 | -1.544 | 18.084 | 0.153 |
|  | 13 | -2.000 | 18.787 | 0.145 | -1.387 | 19.489 | 0.157 |
|  | 14 | -1.877 | 19.416 | 0.144 | -1.370 | 20.179 | 0.155 |
|  | 15 | -1.754 | 20.067 | 0.143 | -1.382 | 20.814 | 0.153 |
|  | 18 | -1.379 | 22.062 | 0.145 | -1.463 | 22.376 | 0.156 |
| **2001 MCS** | 3 | -1.933 | 16.834 | 0.081 | -1.354 | 16.561 | 0.088 |
|  | 5 | -1.933 | 16.282 | 0.090 | -1.743 | 16.160 | 0.093 |
|  | 7 | -1.933 | 16.227 | 0.106 | -1.779 | 16.297 | 0.116 |
|  | 11 | -1.933 | 18.076 | 0.146 | -1.108 | 18.640 | 0.166 |

BMI: Body Mass Index, LMS: Lambda Mu Sigma, NSHD: Medical Research Council National Survey of Health and Development, NCDS National Child Development Study, BCS: British Cohort Study, ALSPAC: Avon Longitudinal Study of Parents and Children, MCS: Millennium Cohort Study
