# Supplementary material for: How Has the Age-Related Process of Overweight or Obesity Development Changed over Time? Co-ordinated Analyses of Individual Participant Data from Five United Kingdom Birth Cohorts
Source: PLoS Med. 2015 May 19;12(5):e1001828. doi: 10.1371/journal.pmed.1001828 (PMC4437909; doi:10.1371/journal.pmed.1001828)
Supplement: S4 Table — (DOCX) [file pmed.1001828.s009.docx]

**S4 Table. Adulthood LMS values at target assessment ages from sex and study stratified models applied to serial BMI data**

|  |  | **Male** | | | **Female** | | |
| --- | --- | --- | --- | --- | --- | --- | --- |
|  | Age | L | M | S | L | M | S |
| **1946 NSHD** | 20 | -0.506 | 22.414 | 0.109 | -1.959 | 21.225 | 0.118 |
|  | 26 | -0.506 | 23.175 | 0.115 | -1.842 | 21.802 | 0.125 |
|  | 36 | -0.506 | 24.444 | 0.125 | -1.619 | 22.980 | 0.140 |
|  | 43 | -0.506 | 23.331 | 0.131 | -1.426 | 24.122 | 0.152 |
|  | 53 | -0.506 | 26.600 | 0.141 | -1.132 | 25.911 | 0.171 |
|  | 63 | -0.506 | 27.868 | 0.151 | -0.854 | 27.431 | 0.188 |
| **1958 NCDS** | 23 | -1.155 | 22.636 | 0.117 | -1.667 | 21.515 | 0.125 |
|  | 33 | -0.500 | 25.053 | 0.134 | -1.060 | 23.591 | 0.166 |
|  | 42 | -0.585 | 26.263 | 0.145 | -1.138 | 24.723 | 0.176 |
|  | 44 | -0.636 | 26.778 | 0.148 | -1.161 | 25.277 | 0.178 |
|  | 50 | -0.717 | 27.554 | 0.154 | -1.152 | 25.900 | 0.182 |
| **1970 BCS** | 26 | -0.835 | 24.055 | 0.132 | -1.617 | 22.546 | 0.146 |
|  | 30 | -0.835 | 25.096 | 0.140 | -1.503 | 23.366 | 0.158 |
|  | 34 | -0.835 | 25.927 | 0.147 | -1.362 | 24.112 | 0.170 |
|  | 42 | -0.835 | 26.830 | 0.156 | -1.141 | 25.178 | 0.188 |

BMI: Body Mass Index, LMS: Lambda Mu Sigma, NSHD: Medical Research Council National Survey of Health and Development, NCDS National Child Development Study, BCS: British Cohort Study
