# Supplementary material for: How Has the Age-Related Process of Overweight or Obesity Development Changed over Time? Co-ordinated Analyses of Individual Participant Data from Five United Kingdom Birth Cohorts
Source: PLoS Med. 2015 May 19;12(5):e1001828. doi: 10.1371/journal.pmed.1001828 (PMC4437909; doi:10.1371/journal.pmed.1001828)
Supplement: S5 Table — (DOCX) [file pmed.1001828.s010.docx]

**S5 Table. Percentages of childhood BMI values above select centiles, estimated from sex and study stratified LMS models**

|  |  | **1946 NSHD** | | **1958 NCDS** | | **1970 BCS** | | **1991 ALSPAC** | | **2001 MCS** | |
| --- | --- | --- | --- | --- | --- | --- | --- | --- | --- | --- | --- |
|  |  | **Male** | **Female** | **Male** | **Female** | **Male** | **Female** | **Male** | **Female** | **Male** | **Female** |
| Centile | Expected % above | Observed % above | | | | | | | | | |
| 98 | 2 | 2.6 | 2.6 | 3.1 | 2.8 | 3.4 | 2.9 | 2.5 | 2.3 | 2.9 | 2.7 |
| 91 | 9 | 8.6 | 7.9 | 8.5 | 9.1 | 9.7 | 10.4 | 10.2 | 9.7 | 9.1 | 9.5 |
| 75 | 25 | 23.3 | 23.8 | 22.4 | 23.6 | 24.5 | 25.9 | 25.0 | 25.1 | 23.7 | 24.3 |
| 50 | 50 | 48.7 | 49.0 | 49.8 | 49.1 | 50.9 | 51.0 | 47.9 | 48.6 | 48.7 | 48.9 |
| 25 | 75 | 74.9 | 75.4 | 77.1 | 76.2 | 78.6 | 77.0 | 74.1 | 73.4 | 75.7 | 75.4 |
| 9 | 91 | 91.1 | 91.2 | 92.4 | 92.0 | 93.0 | 92.7 | 91.2 | 90.9 | 92.1 | 91.9 |
| 2 | 98 | 97.3 | 97.5 | 97.6 | 97.8 | 98.1 | 98.3 | 97.8 | 97.9 | 97.9 | 97.9 |

BMI: Body Mass Index, LMS: Lambda Mu Sigma, NSHD: Medical Research Council National Survey of Health and Development, NCDS National Child Development Study, BCS: British Cohort Study, ALSPAC: Avon Longitudinal Study of Parents and Children, MCS: Millennium Cohort Study
