# Supplementary material for: How Has the Age-Related Process of Overweight or Obesity Development Changed over Time? Co-ordinated Analyses of Individual Participant Data from Five United Kingdom Birth Cohorts
Source: PLoS Med. 2015 May 19;12(5):e1001828. doi: 10.1371/journal.pmed.1001828 (PMC4437909; doi:10.1371/journal.pmed.1001828)
Supplement: S6 Table — (DOCX) [file pmed.1001828.s011.docx]

**S6 Table. Percentages of adulthood BMI values above select centiles, estimated from sex and study stratified LMS models**

|  |  | **1946 NSHD** | | **1958 NCDS** | | **1970 BCS** | |
| --- | --- | --- | --- | --- | --- | --- | --- |
|  |  | **Male** | **Female** | **Male** | **Female** | **Male** | **Female** |
| Centile | Expected % above | Observed % above | | | | | |
| 98 | 2 | 2.7 | 2.6 | 2.6 | 2.8 | 2.3 | 2.8 |
| 91 | 9 | 8.5 | 9.3 | 8.7 | 9.8 | 9.0 | 10.3 |
| 75 | 25 | 25.4 | 24.6 | 23.1 | 24.2 | 24.3 | 25.0 |
| 50 | 50 | 50.3 | 48.7 | 49.2 | 47.8 | 50.4 | 48.2 |
| 25 | 75 | 75.6 | 75.6 | 76.1 | 75.1 | 75.6 | 74.4 |
| 9 | 91 | 91.7 | 91.8 | 91.4 | 91.9 | 91.1 | 92.1 |
| 2 | 98 | 97.7 | 97.6 | 97.7 | 98.1 | 97.8 | 98.0 |

BMI: Body Mass Index, LMS: Lambda Mu Sigma, NSHD: Medical Research Council National Survey of Health and Development, NCDS National Child Development Study, BCS: British Cohort Study
