# Supplementary material for: How Has the Age-Related Process of Overweight or Obesity Development Changed over Time? Co-ordinated Analyses of Individual Participant Data from Five United Kingdom Birth Cohorts
Source: PLoS Med. 2015 May 19;12(5):e1001828. doi: 10.1371/journal.pmed.1001828 (PMC4437909; doi:10.1371/journal.pmed.1001828)
Supplement: S7 Table — (DOCX) [file pmed.1001828.s012.docx]

**S7 Table. Age scale and EDFs used in the fitting of sex, study, and life course stage stratified LMS models applied to serial BMI data**

|  | **Male** | | |  | **Female** | | |  |
| --- | --- | --- | --- | --- | --- | --- | --- | --- |
|  | Age scale | EDF for L | EDF for M | EDF for S | Age | EDF for L | EDF for M | EDF for S |
| **1946 NSHD** |  |  |  |  |  |  |  |  |
| Childhood | Original | 4 | 5 | 4 | Original | 4 | 5 | 4 |
| Adulthood | Original | 1 | 2 | 2 | Rescaled | 2 | 3 | 2 |
| **1958 NCDS** |  |  |  |  |  |  |  |  |
| Childhood | Original | 3 | 3 | 3 | Rescaled | 3 | 3 | 3 |
| Adulthood | Rescaled | 3 | 4 | 2 | Rescaled | 3 | 4 | 3 |
| **1970 BCS** |  |  |  |  |  |  |  |  |
| Childhood | Original | 1 | 2 | 2 | Original | 2 | 2 | 2 |
| Adulthood | Original | 1 | 3 | 2 | Rescaled | 2 | 3 | 2 |
| **1991 ALSPAC** |  |  |  |  |  |  |  |  |
| Childhood | Rescaled | 2 | 3 | 4 | Rescaled | 3 | 4 | 4 |
| **2001 MCS** |  |  |  |  |  |  |  |  |
| Childhood | Original | 1 | 4 | 3 | Original | 3 | 4 | 4 |

BMI: Body Mass Index, EDF: Equivalent Degrees of Freedom, LMS: Lambda Mu Sigma, NSHD: Medical Research Council National Survey of Health and Development, NCDS National Child Development Study, BCS: British Cohort Study, ALSPAC: Avon Longitudinal Study of Parents and Children, MCS: Millennium Cohort Study
