# Supplementary material for: How Has the Age-Related Process of Overweight or Obesity Development Changed over Time? Co-ordinated Analyses of Individual Participant Data from Five United Kingdom Birth Cohorts
Source: PLoS Med. 2015 May 19;12(5):e1001828. doi: 10.1371/journal.pmed.1001828 (PMC4437909; doi:10.1371/journal.pmed.1001828)
Supplement: S8 Table — (DOCX) [file pmed.1001828.s013.docx]

**S8 Table. Study stratified binary logistic multilevel models in males to describe overweight or obesity (versus normal weight) as a restricted cubic spline of age**

|  | **1946 NSHD** | **1958 NCDS** | **1970 BCS** | **1991 ALSPAC** | **2001 MCS** |
| --- | --- | --- | --- | --- | --- |
|  | Estimate (95% Confidence Interval) | | | | |
| Fixed effect |  |  |  |  |  |
| Intercept | -9.094 (-9.696, -8.492) | -2.439 (-2.664, -2.215) | -1.405 (-1.762, -1.048) | -1.251 (-1.596, -0.905) | -1.679 (-1.772, -1.586) |
| 1^st^ spline term | -0.447 (-0.481, -0.413) | -0.002 (-0.016, 0.012) | 0.073 (0.047, 0.099) | 0.159 (0.059, 0.259) | -0.163 (-0.199, -0.128) |
| 2^nd^ spline term | 11.672 (10.511, 12.832) | 0.564 (0.493, 0.635) | 0.377 (0.258, 0.497) | 0.343 (-0.563, 1.249) | 0.334 (0.271, 0.397) |
| 3^rd^ spline term | -17.358 (-19.234, -15.483) | -1.002 (-1.126, -0.879) | -0.781 (-0.993, -0.568) | -3.522 (-6.914, -0.129) | -- |
| 4^th^ spline term | 5.542 (4.750, 6.334) | -- | -- | 5.771 (2.150, 9.392) | -- |
| Random effect variance |  |  |  |  |  |
| Intercept | 6.189 (4.722, 7.657) | 1.861 (1.721, 2.001) | 1.534 (1.425, 1.643) | 3.462 (3.255, 3.668) | 1.868 (1.742, 1.993) |
| 1^st^ spline term | 0.010 (0.005, 0.015) | -- | -- | -- | -- |
| 2^nd^ spline term | 0.141 (0.009, 0.274) | -- | -- | -- | -- |
| 3^rd^ spline term | -- | 0.003 (0.002, 0.005) | -- | -- | -- |
| 4^th^ spline term | 0.274 (-0.188, 0.736) | -- | -- | -- | -- |
| Random effect covariance |  |  |  |  |  |
| Intercept, 1^st^ spline term | 0.241 (0.156, 0.326) | -- | -- | -- | -- |
| Intercept, 2^nd^ spline term | -1.045 (-1.476, -0.614) | -- | -- | -- | -- |
| Intercept, 3^rd^ spline term | -- | -0.016 (-0.027, -0.005) | -- | -- | -- |
| Intercept, 4^th^ spline term | 1.764 (0.965, 2.562) | -- | -- | -- | -- |
| 1^st^ spline term, 2^nd^ spline term | -0.037 (-0.063, -0.012) | -- | -- | -- | -- |
| 1^st^ spline term, 3^rd^ spline term | -- | -- | -- | -- | -- |
| 1^st^ spline term, 4^th^ spline term | 0.059 (0.012, 0.107) | -- | -- | -- | -- |
| 2^nd^ spline term, 3^rd^ spline term | -- | -- | -- | -- | -- |
| 2^nd^ spline term, 4^th^ spline term | -0.207 (-0.454, 0.040) | -- | -- | -- | -- |
| 3^rd^ spline term, 4^th^ spline term | -- | -- | -- | -- | -- |
| Location of intercept (years) | 21.334 | 27.146 | 26.265 | 11.542 | 6.522 |
| Location of knots (years) |  |  |  |  |  |
| 1^st^ | -19.334 | -19.894 | -16.162 | -4.090 | -3.477 |
| 2^nd^ | -15.167 | -11.330 | -9.241 | -1.939 | -0.989 |
| 3^rd^ | -6.834 | 6.294 | 4.002 | -0.478 | 4.725 |
| 4^th^ | 14.749 | 23.526 | 16.336 | 1.577 | -- |
| 5^th^ | 39.249 | -- | -- | 6.041 | -- |

NSHD: Medical Research Council National Survey of Health and Development, NCDS National Child Development Study, BCS: British Cohort Study, ALSPAC: Avon Longitudinal Study of Parents and Children, MCS: Millennium Cohort Study
