# Supplementary material for: How Has the Age-Related Process of Overweight or Obesity Development Changed over Time? Co-ordinated Analyses of Individual Participant Data from Five United Kingdom Birth Cohorts
Source: PLoS Med. 2015 May 19;12(5):e1001828. doi: 10.1371/journal.pmed.1001828 (PMC4437909; doi:10.1371/journal.pmed.1001828)
Supplement: S9 Table — (DOCX) [file pmed.1001828.s014.docx]

**S9 Table. Study stratified binary logistic multilevel models in females to describe overweight or obesity (versus normal weight) as a restricted cubic spline of age**

|  | **1946 NSHD** | **1958 NCDS** | **1970 BCS** | **1991 ALSPAC** | **2001 MCS** |
| --- | --- | --- | --- | --- | --- |
|  | Estimate (95% Confidence Interval) | | | | |
| Fixed effect |  |  |  |  |  |
| Intercept | -10.134 (-10.996, -9.272) | -2.453 (-2.678, -2.228) | -1.523 (-1.710, -1.335) | -0.731 (-1.017, -0.446) | -1.241 (-1.329, -1.152) |
| 1^st^ spline term | -0.478 (-0.524, -0.433) | -0.023 (-0.037, -0.010) | 0.031 (0.016, 0.045) | 0.175 (0.096, 0.255) | -0.054 (-0.088, -0.020) |
| 2^nd^ spline term | 19.585 (16.700, 22.470) | 0.402 (0.335, 0.470) | 0.110 (0.076, 0.144) | -0.567 (-1.211, 0.077) | 0.167 (0.107, 0.227) |
| 3^rd^ spline term | -38.931 (-45.492, -32.370) | -0.671 (-0.789, -0.554) | -1.048 (-1.350, -0.746) | 0.421 (-1.721, 2.564) | -- |
| 4^th^ spline term | 21.300 (16.763, 25.837) | -- | -- | 1.541 (-0.880, 3.963) | -- |
| 5^th^ spline term | -2.439 (-3.513, -1.364) | -- | -- | -- | -- |
| Random effect variance |  |  |  |  |  |
| Intercept | 1.519 (1.364, 1.674) | 2.051 (1.907, 2.195) | 1.728 (1.619, 1.836) | 3.047 (2.868, 3.225) | 1.815 (1.696, 1.934) |
| 1^st^ spline term | -- | -- | -- | -- | -- |
| 2^nd^ spline term | -- | -- | -- | -- | -- |
| 3^rd^ spline term | -- | 0.002 (0.001, 0.003) | -- | -- | -- |
| 4^th^ spline term | 0.002 (0.001, 0.003) | -- | -- | -- | -- |
| 5^th^ spline term | -- | -- | -- | -- | -- |
| Random effect covariance |  |  |  |  |  |
| Intercept, 1^st^ spline term | -- | -- | -- | -- | -- |
| Intercept, 2^nd^ spline term | -- | -- | -- | -- | -- |
| Intercept, 3^rd^ spline term | -- | -0.026 (-0.036, -0.016) | -- | -- | -- |
| Intercept, 4^th^ spline term | -0.023 (-0.034, -0.018) | -- | -- | -- | -- |
| Intercept, 5^th^ spline term | -- | -- | -- | -- | -- |
| 1^st^ spline term, 2^nd^ spline term | -- | -- | -- | -- | -- |
| 1^st^ spline term, 3^rd^ spline term | -- | -- | -- | -- | -- |
| 1^st^ spline term, 4^th^ spline term | -- | -- | -- | -- | -- |
| 1^st^ spline term, 5^th^ spline term | -- | -- | -- | -- | -- |
| 2^nd^ spline term, 3^rd^ spline term | -- | -- | -- | -- | -- |
| 2^nd^ spline term, 4^th^ spline term | -- | -- | -- | -- | -- |
| 2^nd^ spline term, 5^th^ spline term | -- | -- | -- | -- | -- |
| 3^rd^ spline term, 4^th^ spline term | -- | -- | -- | -- | -- |
| 3^rd^ spline term, 5^th^ spline term | -- | -- | -- | -- | -- |
| 4^th^ spline term, 5^th^ spline term | -- | -- | -- | -- | -- |
| Location of intercept (years) | 22.395 | 27.610 | 26.440 | 11.725 | 6.509 |
| Location of knots (years) |  |  |  |  |  |
| 1^st^ | -20.395 | -20.358 | -16.315 | -4.267 | -3.461 |
| 2^nd^ | -16.311 | -11.747 | -0.440 | -2.045 | -0.988 |
| 3^rd^ | -11.561 | 6.018 | 3.633 | -0.202 | 4.659 |
| 4^th^ | -2.095 | 23.062 | 16.090 | 1.986 | -- |
| 5^th^ | 14.105 | -- | -- | 5.941 | -- |
| 6^th^ | 40.105 | -- | -- | -- | -- |

NSHD: Medical Research Council National Survey of Health and Development, NCDS National Child Development Study, BCS: British Cohort Study, ALSPAC: Avon Longitudinal Study of Parents and Children, MCS: Millennium Cohort Study
