# Supplementary material for: How Has the Age-Related Process of Overweight or Obesity Development Changed over Time? Co-ordinated Analyses of Individual Participant Data from Five United Kingdom Birth Cohorts
Source: PLoS Med. 2015 May 19;12(5):e1001828. doi: 10.1371/journal.pmed.1001828 (PMC4437909; doi:10.1371/journal.pmed.1001828)
Supplement: S10 Table — (DOCX) [file pmed.1001828.s015.docx]

**S10 Table. Kruskal-Wallis tests of between-study differences in height, weight, and BMI Z-scores according to the UK-WHO chart at 10 or 11 years of age; diagonal values are medians (IQR) and off-diagonal values are Bonferroni corrected p-values**

|  | **1946 NSHD** | **1958 NCDS** | **1970 BCS** | **1991 ALSPAC** | **2001 MCS** |
| --- | --- | --- | --- | --- | --- |
| **Height** |  |  |  |  |  |
| **NSHD 1946** | -0.44 (-0.94, 0.34) |  |  |  |  |
| **NCDS 1958** | <0.001 | -0.24 (-0.90, 0.45) |  |  |  |
| **BCS 1970** | <0.001 | >0.999 | -0.22 (-0.84, 0.44) |  |  |
| **ALSPAC 1991** | <0.001 | <0.001 | <0.001 | 0.39 (-0.27, 1.07) |  |
| **MCS 2001** | <0.001 | <0.001 | <0.001 | <0.001 | 0.21 (-0.44, 0.91) |
| **Weight** |  |  |  |  |  |
| **NSHD 1946** | -0.20 (-0.84, 0.43) |  |  |  |  |
| **NCDS 1958** | >0.999 | -0.22 (-0.86, 0.47) |  |  |  |
| **BCS 1970** | 0.488 | 0.064 | -0.17 (-0.78, 0.45) |  |  |
| **ALSPAC 1991** | <0.001 | <0.001 | <0.001 | 0.46 (-0.27, 1.23) |  |
| **MCS 2001** | <0.001 | <0.001 | <0.001 | 0.295 | 0.52 (-0.22, 1.29) |
| **BMI** |  |  |  |  |  |
| **NSHD 1946** | -0.05 (-0.72, 0.59) |  |  |  |  |
| **NCDS 1958** | <0.001 | -0.22 (-0.86, 0.50) |  |  |  |
| **BCS 1970** | 0.103 | <0.001 | -0.11 (-0.72, 0.54) |  |  |
| **ALSPAC 1991** | <0.001 | <0.001 | <0.001 | 0.31 (-0.50, 1.21) |  |
| **MCS 2001** | <0.001 | <0.001 | <0.001 | <0.001 | 0.50 (-0.31, 1.40) |

BMI: Body Mass Index, IQR: Inter-Quartile Range, UK-WHO: United Kingdom-World Health Organisation, NSHD: Medical Research Council National Survey of Health and Development, NCDS National Child Development Study, BCS: British Cohort Study, ALSPAC: Avon Longitudinal Study of Parents and Children, MCS: Millennium Cohort Study
